# Linear-Scaling Open-Shell MP2 Approach: Algorithm, Benchmarks, and Large-Scale Applications

**DOI:** 10.1021/acs.jctc.1c00093

**Published:** 2021-04-05

**Authors:** P. Bernát Szabó, József Csóka, Mihály Kállay, Péter R. Nagy

**Affiliations:** Department of Physical Chemistry and Materials Science, Budapest University of Technology and Economics, P.O. Box 91, H-1521 Budapest, Hungary

## Abstract

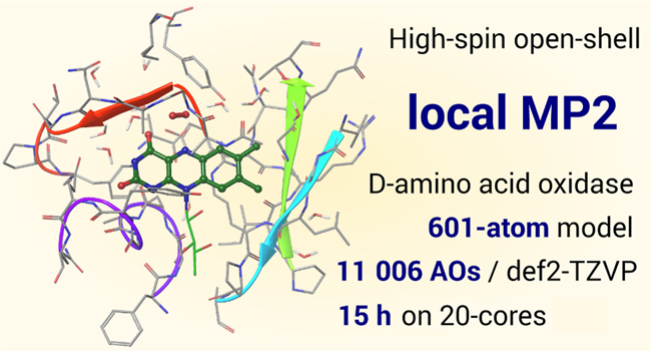

A linear-scaling local second-order Møller–Plesset (MP2) method is presented for high-spin open-shell molecules based on restricted open-shell (RO) reference functions. The open-shell local MP2 (LMP2) approach inherits the iteration- and redundancy-free formulation and the completely integral-direct, OpenMP-parallel, and memory and disk use economic algorithms of our closed-shell LMP2 implementation. By utilizing restricted local molecular orbitals for the demanding integral transformation step and by introducing a novel long-range spin-polarization approximation, the computational cost of RO-LMP2 approaches that of closed-shell LMP2. Extensive benchmarks were performed for reactions of radicals, ionization potentials, as well as spin-state splittings of carbenes and transition-metal complexes. Compared to the conventional MP2 reference for systems of up to 175 atoms, local errors of at most 0.1 kcal/mol were found, which are well below the intrinsic accuracy of MP2. RO-LMP2 computations are presented for challenging protein models of up to 601 atoms and 11 000 basis functions, which involve either spin states of a complexed iron ion or a highly delocalized singly occupied orbital. The corresponding runtimes of 9–15 h obtained with a single, many-core CPU demonstrate that MP2, as well as spin-scaled MP2 and double-hybrid density functional methods, become widely accessible for open-shell systems of unprecedented size and complexity.

## 1 Introduction

While open-shell species are ubiquitous in chemistry, their investigation remains challenging from both the experimental and the theoretical perspective.^1^ Here, we focus on systems of a high-spin open-shell electronic structure, for which single-reference quantum chemical methods can provide an accurate description comparable to what is expected for closed-shell molecules.^2,3^ The variety of such systems includes the ionized and electron-attached states of closed-shell molecules; species relevant in combustion, polymer, atmospheric, interstellar, electro-, and redox chemistry; and radicals appearing as intermediates or transition states of reactions.^1^

The importance and difficulties of the explicit treatment of electron correlation for such systems and the related processes are also well understood.^2,4,5^ The second-order Møller–Plesset approach (MP2)^6^ is one of the standard tools for that purpose. While the accuracy of conventional MP2 does not reach that of the “gold standard” coupled-cluster (CC) model with single, double, and perturbative triple excitations [CCSD(T)],^7,8^ its favorable computational cost motivated the development of various improved MP2-based methods.^9,10^ Among those, the spin-component-scaled (SCS) MP2 schemes^11−15^ and the double-hybrid (DH) density functionals,^16−19^ both proposed by Grimme, have gained wide popularity.^15,18^ In the SCS-MP2 methods, the opposite- and same-spin contributions to the correlation energy are scaled by different empirical factors, whereas for the DH functionals, the energy is augmented with an MP2-like second-order perturbation theory (PT2) correction evaluated on Kohn–Sham (KS) orbitals. The introduction of spin-scaled PT2 expressions into the formulation of DH functionals^20−24^ turned out to be particularly successful to raise the accuracy of DH functionals above that of conventional density functionals.^21,24−26^

Especially when combined with the resolution-of-identity or density-fitting (DF) technique,^27^ MP2-based methods can target systems of more than 100 atoms,^9^ thereby extending the about 30-atom applicability limit of CCSD(T)^28^ considerably. The Laplace transform (LT) technique^29,30^ proposed by Almlöf to eliminate the energy denominator of MP2 has also become fundamental to reduce the fifth-power-scaling computational complexity of MP2.^31−39^ Aiming toward the same goal, the particularly simple form of MP2 was also utilized in a number of creative developments on the basis of, for instance, Cholesky-decomposed pseudo-density matrices;^40−43^ stochastic,^44,45^ quadrature-based,^46^ and pseudospectral^47,48^ approaches; nonorthogonal^49,50^ or Slater-type orbitals;^51,52^ tensor hypercontraction;^53,54^ as well as large-scale parallelization.^35,55,56^

Parallel to such reformulations, the group of local correlation approaches^57−59^ exploits the rapid decay of electron correlation with distance, especially in combination with localized molecular orbitals (LMOs). Following the pioneering work of Pulay and Saebø,^36,60^ the correlation energy contribution of distant LMO pairs can be approximated via a more cost-efficient level of theory (pair approximation), often using a restricted, spatially close list of correlating orbitals (domain approximation). Particular methods also compute the first-order MP (MP1) amplitudes, required for the MP2 energy, directly in the LMO basis, for which the solution of coupled amplitude equations is frequently accelerated using some sort of MP2-based natural orbitals (NOs).^61−67^ Alternatively, the coupling of MP1 amplitudes with distant LMO indices can be neglected using fragmentation approximations^57,58,68−87^ and fragment-specific semicanonical basis sets. Our previous developments^38,88−90^ combine the above benefits of decoupled MP1 amplitude expressions with the sparsity provided by the LMO basis via an LT or Cholesky-decomposition (CD)-based MP2 formulation.^29,30,91^ We also utilize a form of MP2-based local NOs (LNOs) in our LNO-CCSD(T)^89,90,92^ and higher-order LNO-CC^90,93^ schemes. However, as the computational cost of the MP2-based LNO construction is comparable to that of the MP2 correlation energy, our local MP2 (LMP2) approach employs only the pair and domain approximations in combination with an LT/CD-based energy expression written in the LMO basis but does not require NOs.^38,88−90^

Compared to the variety of local correlation methods targeting closed-shell systems, the application of local approximations for open-shell cases is much less explored. Open-shell extensions of the incremental scheme^78^ were developed by Dolg, Tew, Friedrich, and co-workers based on unrestricted Hartree–Fock (UHF)^86^ as well as restricted open-shell HF (ROHF)^87^ references. Most recently, the high-spin open-shell variants of the pair NO (PNO)-based method of Werner, Ma, and co-workers,^65,94,95^ as well as the domain-based local PNO (DLPNO) method of Neese, Valeev, Hansen, Saitow, Guo, Kumar, and co-workers^63,96−98^ were also published up to the CCSD(T) level of theory. The DLPNO family of methods employs the multireference second-order Ansatz of the *n*-electron valence state perturbation theory (NEVPT2)^99^ for the PNO generation,^63^ while the PNO methods of Werner and Ma utilize a spin-adapted MP2 formulation (PNO-RMP2).^65^ Both approaches share the benefit of spin-free amplitudes useful to obtain a spin-restricted set of PNOs at the price of a somewhat more complicated second-order treatment.

Since neither NOs nor iterative amplitude equations are required for the efficient computation of MP1 amplitudes in our LMP2 approach,^38,89^ we prioritized simplicity and chose the ROHF-based but unrestricted MP2 Ansatz proposed by Lauderdale and Bartlett^100^ and Knowles et al.^101^ However, the most demanding integral transformation step is carried out in a restricted, intermediate MO basis; thus, the computational cost remains comparable to that of the parent closed-shell LMP2 method. To that end, the use of ROHF or restricted open-shell KS (ROKS) reference is required, but UHF and unrestricted KS (UKS) orbitals are also supported by the construction of quasi-restricted orbitals (QROs).^102^

For the generalization of our local correlation methods to the high-spin open-shell case, here, we identify and resolve a number of technical subtleties emerging already at the LMP2 level of theory. Special attention is devoted to the treatment of singly occupied MOs (SOMOs) in the pair and domain approximations as well as in the pair and domain correlation energy contributions and to the energy contribution of single excitations. The independent evaluation of the MP1 amplitudes also allows for the introduction of a novel cost-reduction approach: we show that up to 50–90% of the correlation energy contributions can be computed relying on closed-shell algorithms by approximating long-range spin-polarization effects far away from the localized SOMOs.

The resulting open-shell LMP2 correlation energies are equivalent to the closed-shell ones for systems with only doubly occupied orbitals in the zeroth-order wave function. The open-shell LMP2 approach inherits the beneficial properties of our previous algorithms,^38,89,90^ which are the iteration- and redundancy-free amplitude evaluation, and the operation-count and memory-efficient, integral-direct, practically disk I/O-free, and OpenMP-parallel implementation. The present local approximations are free from empirical parameters, manual fragment definitions, real-space cutoffs, etc. often associated with local correlation methods. All approximations are systematically improvable and automatically adapt to the electronic structure because of the employed energy and orbital coefficient-based threshold definitions. Additional unique properties include the treatment of near-linear-dependent AO basis sets, integration to multilevel local correlation methods,^103,104^ the utilization of general point group symmetry, and frequent checkpointing.

The accuracy of the open-shell LMP2 method is benchmarked for radical stabilization energies (RSEs), ionization potentials (IPs), and spin-state energy differences of a large set of open-shell species. Mean (maximum) absolute errors against canonical DF-MP2 references are well below the intrinsic accuracy of MP2 being 0.01–0.06 (0.04–0.13) kcal/mol for all three types of quantities with various basis sets. The same errors remain in the 0.01–0.12 kcal/mol range for a smaller set of systems including 37–175 atoms, while the corresponding LMP2 calculations take only up to 3–4 h with an 8-core CPU.

The capabilities of the present open-shell LMP2 code are demonstrated on three-dimensional, real-life protein models including 565 and 601 atoms and about 11 000 atomic orbitals. Both examples represent current challenges of large-scale correlated calculations (see Section 4): the lowest-lying quintet and triplet states of the first system were especially complicated to find at the self-consistent field (SCF) level, and one of the SOMOs of the other molecule is delocalized over a large fragment, leading to extremely large domains to handle. In spite of these difficulties, it was feasible to perform LMP2 computations for four species in this size range, each taking about 9–15 h on a single, 20-core CPU. Thus, the present implementation is highly capable of extending the reach of open-shell (spin-scaled) MP2 and DH density functional computations to systems of unprecedented size.

The paper is organized as follows. Sections 2 and 3 provide the theoretical details of the LMP2 Ansatz and the corresponding algorithms, respectively. The employed technical details and test systems are introduced in Section 4. The accuracy of the individual and combined local approximations is assessed in Sections 5 and 6. Finally, Section 7 presents large-scale applications and analyzes the corresponding computational requirements.

## 2 Theoretical Background

A restricted open-shell reference determinant of doubly and singly occupied molecular orbitals (DOMOs and SOMOs, respectively) is assumed. These orbitals will be subjected to various orbital transformations, and the notations distinguishing the different orbital sets are collected in Table 1. The correlation energy expressions of the conventional theory will be introduced in terms of unrestricted, semicanonical (also known as pseudocanonical) MOs denoted by (*i*, *j*, *k*, ..., *I*, *J*, *K*, ...) and (*a*, *b*, *c*, ..., *A*, *B*, *C*, ...) indices for the occupied and virtual subsets, respectively. Lower (upper) case indices label the spin-up (spin-down) set of semicanonical orbitals. Local approximations will rely on localized molecular orbitals (LMOs) obtained from a restricted open-shell reference , while these LMOs will be labeled as *i*′, *j*′, *k*′, ... (*I*′, *J*′, *K*′, ...), respectively, when occupied by spin-up (spin-down) electrons.

**Table 1:** Summary of Index Notations for Orbital Sets Employed in Sections 2 and 3

*i*, *j*, *k*, ...	(semi-)canonical occupied orbitals (spin-up)
*I*, *J*, *K*, ...	(semi-)canonical occupied orbitals (spin-down)
*a*, *b*, *c*, ...	(semi-)canonical virtual orbitals (spin-up)
*A*, *B*, *C*, ...	(semi-)canonical virtual orbitals (spin-down)
*i*′, *j*′, *k*′, ...	localized restricted occupied orbitals (spin-up)
*I*′, *J*′, *K*′, ...	localized restricted occupied orbitals (spin-down)
	localized restricted occupied orbitals (spatial)
*ĩ*, ..., *ã*, ...	(semi-)canonical orbitals in the primary or extended domain (spin-up)
*Ĩ*, ..., *Ã*, ...	(semi-)canonical orbitals in the primary or extended domain (spin-down)
μ, ν, λ, ...	atomic orbitals
*P*, *Q*, ...	auxiliary functions for the DF approximation

### 2.1 Canonical Open-Shell MP2 Ansatz

Here, we briefly summarize the MP2 approach introduced by Lauderdale and Bartlett^100^ and Knowles et al.^101^ for restricted open-shell reference determinants. Starting from a set of restricted orbitals, spin-up and spin-down Fock matrices are constructed using the DOMOs and SOMOs for the former and only the DOMOs for the latter. The spin-up (spin-down) MOs of the restricted open-shell determinant are then canonicalized separately using the respective spin-up (spin-down) Fock matrices, yielding the semicanonicalized MO sets. The corresponding second-order correlation energy (*E*_MP2_^c^) is calculated relying on an unrestricted formalism
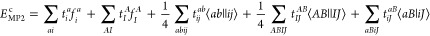
1Here, quantities *t*_*i*_^*a*^ and *t*_*ij*_^*ab*^ ... denote MP1 amplitudes corresponding to single and double excitations. Moreover, *f*_*i*_^*a*^ and ⟨*ab*|*ij*⟩ stand for Fock-matrix elements and electron repulsion integrals (ERIs) in the Dirac notation, respectively, while ⟨*ab*∥*ij*⟩ = ⟨*ab*|*ij*⟩ – ⟨*ab*|*ji*⟩. The beneficial properties of this correlation energy expression include the invariance to the separate unitary transformation of the occupied and unoccupied MOs. This opens the possibility of introducing local correlation approximations exploiting LMOs. Naturally, eq 1 is equivalent to the closed-shell MP2 correlation energy in the special case of closed-shell systems.

### 2.2 Open-Shell Local MP2 Ansatz

The present open-shell local MP2 Ansatz is constructed analogously to our highly efficient closed-shell local MP2 (LMP2) implementation.^38,88,89^ The LMP2 approach employs ideas from fragmentation approaches, such as the incremental expansion^78,86,87^ up to orbital pairs, which can also be interpreted as pair approximations for distant orbital pairs as employed frequently in direct local correlation approaches.^36,60−62,64,67^ The main correlation energy contribution is obtained using orbital-specific basis sets reminiscent of the cluster-in-molecule,^76,80,82^ as well as the divide-expand-consolidate^84,85^ models. Moreover, a Laplace-transform (LT) or Cholesky-decomposition (CD)-based MP2 formulation^29,30,38,91^ is employed to circumvent redundant amplitude evaluations and the need for the iterative solution of MP1 amplitude equations expressed in a noncanonical basis.

By exploiting the unitary invariance of eq 1, *E*_MP2_^c^ can be rewritten in terms of restricted orbitals. Then, *E*_MP2_^c^ is expressed in terms of correlation energy contributions of occupied orbitals by separating one occupied index in the summations of eq 1
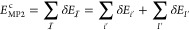
2It is important to emphasize that  denotes a spatial orbital occupied by either one or two electrons, while *i*′ (*I*′) refers to orbitals with the same spatial component as  but occupied by at most one spin-up (spin-down) electron. Here, we also assume that the restricted orbitals of eq 2 are LMOs; hence, the orbital indices are primed. The equivalence of eqs 1 and 2 can be utilized to define the correlation energy contributions of individual LMOs occupied by spin-up and spin-down electrons
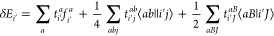
3
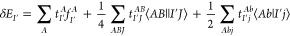
4Note that the last term of eq 1 including both spin-up and spin-down occupied indices results in both the spin-up and spin-down correlation energy contributions (cf. the last terms of eqs 3 and 4) because both of its occupied indices can be transformed to the LMO basis. Furthermore, because of the unitary transformation of *i* → *i*′ (*I* → *I*′) and the restriction of the summations over this index, the complete permutational symmetry of the MP1 amplitudes and two-electron integrals is lost. Consequently, the final terms of eqs 3 and 4 cannot be combined into a single term like in the conventional theory, which explains the appearance of the additional, sixth type of term (third one of eq 4). Note also that the δ*E*_*I*′_ contribution of a singly occupied (SO) restricted LMO is zero by definition; therefore, the correlation energy contribution of the SO LMOs contains only half as many terms as for a doubly occupied (DO) LMO.

To achieve asymptotically linear scaling with system size, the summations of eqs 3 and 4 are restricted. Therefore, the correlation energy contribution of each LMO can be computed with asymptotically constant cost, at least for nonmetallic systems, where the pair correlation energy of all orbital pairs decays with distance. First, a list of occupied and virtual orbitals called the extended domain (ED) is assembled around each LMO, in which the selected LMO serves as the central MO (CMO) of the ED. The occupied subspace, that is, the strong pair LMO list, of the ED consists of those restricted LMOs that have a sufficiently high pair correlation energy with the CMO. Then, the virtual subspace of the ED is constructed from such restricted projected atomic orbitals (PAOs, as defined by eq 6) that are important for the correlation energy contributions of any of the CMO’s strong pairs.

For the accurate and efficient estimation of the MP2 pair correlation energies required for the strong pair list construction, we combine domain approximations with the multipole expansion of the two-electron integrals.^38^ Specifically, the distant and strong pairs are characterized using multipole approximated pair energies evaluated in the primary domains (PDs) of the LMOs forming the pair. Moreover, the pair correlation energies of the distant pairs are used to estimate their contribution to the total MP2 correlation energy
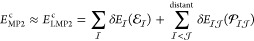
5Here,  and  indicate that the corresponding energy contribution is evaluated only in the orbital spaces of the pair’s PDs or that of the ED, respectively. For closed-shell systems, the resulting expression is equivalent to our spin-restricted LMP2 formulation.^38^

## 3 Local MP2 Algorithm

**Figure 1:**
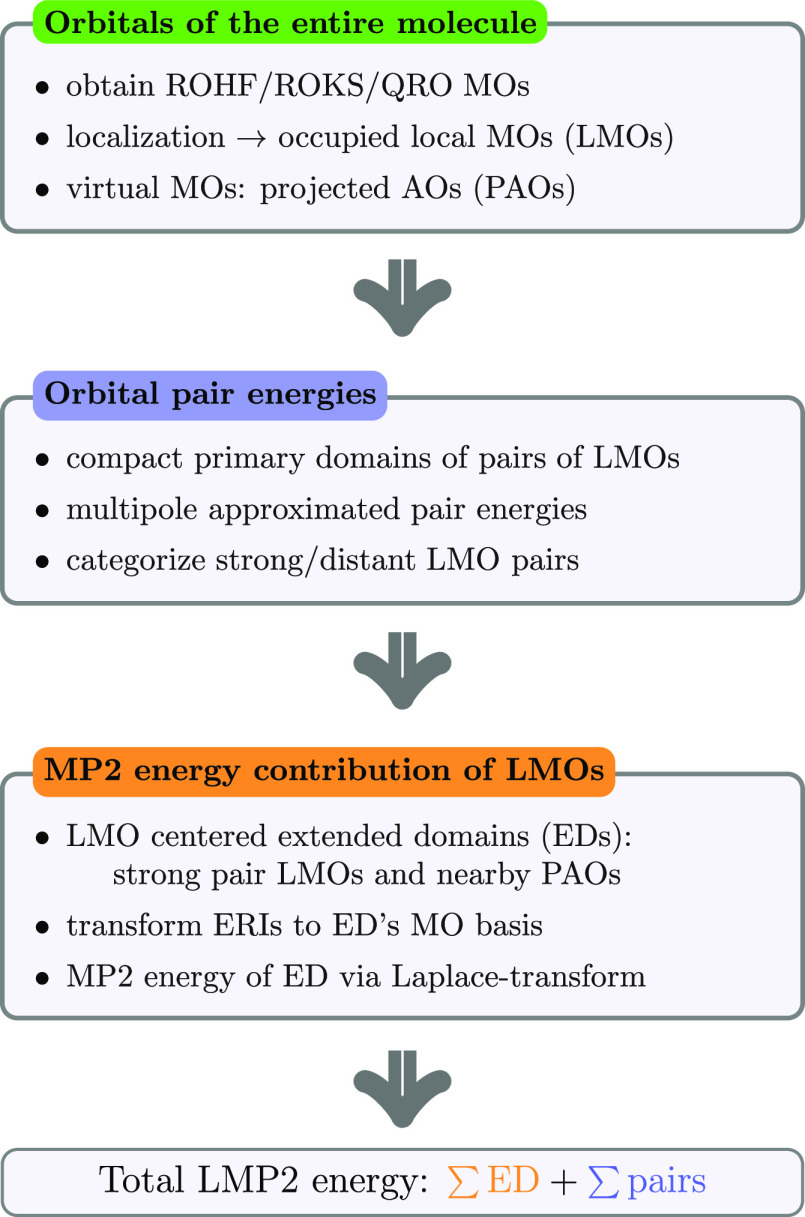
Major algorithmic steps of the presented restricted open-shell LMP2 algorithm.

The major steps of the present restricted open-shell LMP2 (RO-LMP2) algorithm are collected in Figure 1 and discussed in this section in detail.

### 3.1 Self-Consistent Field Calculation

First, a restricted or quasi-restricted open-shell HF or KS reference determinant is obtained for the entire molecule. Then, semicanonical Fock matrices are computed in the AO basis using the (quasi-)restricted electron densities. If any core orbitals are kept frozen, that is, left out of the correlation calculation, then the mixing of those orbitals with the correlated orbitals is avoided during both the semicanonicalization and orbital localization steps.

For the cases where the convergence of ROHF/ROKS is problematic, we also implemented quasi-restricted orbitals (QROs) as proposed by Neese.^102^ Here, the starting point can be an unrestricted HF/KS (UHF/UKS) solution, which is often simpler to find than the ROHF/ROKS one. However, such UHF/UKS solutions may exhibit considerable spin-contamination, that is, the single determinant wave function does not provide the appropriate *S*(*S* + 1) eigenvalue for the square of the spin operator, *Ŝ*^2^, with *S* as the spin quantum number. To circumvent this, the QROs are constructed as the eigenvectors of the total density matrix of the unrestricted computation.^102^ The orbitals obtained in this way with occupation numbers close to 2, 1, and 0 are selected to be DO, SO, and unoccupied in the QRO determinant, respectively, which becomes an eigenfunction of *Ŝ*^2^ by construction. Our numerical experience to date is in line with the findings of Neese and co-workers^63,102^ that the QRO determinant provides a reliable reference (when the RO solution is unavailable) with a somewhat higher energy than the corresponding ROHF/ROKS determinant.

At the few-hundred-atom range, the fourth-power-scaling HF computation can become a computational bottleneck even in combination with the DF approach. However, it is possible to accelerate the evaluation of the exchange term in DF-HF via local DF (LDF) domains, that is, using a restricted list of auxiliary functions for each LMO lying in its proximity.^38,105^ This LDF approach was extended to both restricted open-shell and unrestricted HF- and KS-SCF in the present work. Additionally, the third-power-scaling of exchange evaluation in the LDF-HF approach can be brought down to asymptotically linear scaling by also restricting the list of AOs appearing in the exchange matrix contribution of each LMO.^106,107^ Most recently, our (L)DF-HF algorithms were further sped up by multipole approximations^107^ as well as approximate SCF iterations followed by first-order corrections;^108^ however, these improved schemes were not yet employed here.

### 3.2 Orbital Localization

The localization of the reference (quasi-)restricted occupied orbitals can be carried out by the Boys^109^ or the Pipek–Mezey^110^ scheme, although we found the Boys orbitals to be considerably more suitable for our algorithm.^38^ The highly demanding localization of the unoccupied orbitals is not required. Additionally, the localization can be carried out in a spin-unrestricted or a spin-restricted manner. To take advantage of the computational savings offered by the above RO-LMP2 Ansatz, here we localize the (restricted and correlated) DO and SO orbitals separately. A drawback of this approach may emerge when there is only one SOMO in the entire molecule (or when SOMOs cannot mix due to symmetry) because in this case the unchanged SOMO(s) of the canonical basis may remain considerably delocalized. Moreover, the number of SOMOs is anyway smaller than that of the DOMOs leading to fewer degrees of freedom for their localization and consequently a somewhat larger spread of the localized SOMOs. Alternatively, the spin-up and spin-down orbitals can be localized separately, leading to potentially better localized but unrestricted LMOs and twice as many integrals to transform. Thus, in agreement with previous studies,^57,63,65^ restricted LMOs are employed.

### 3.3 PAO Construction

The LMO optimization is followed by the construction of PAOs,^60,65^ which span the virtual subspace of the entire system defined by
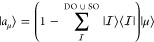
6Here, |*a*_μ_⟩ denotes the PAO projected from AO |μ⟩, and the summation runs over both DO and SO MOs. The PAOs of eq 6 span only the virtual subspace of spin-up electrons because the projection makes the PAOs orthogonal also to the SO subspace. Therefore, the unoccupied subspace of the spin-down electrons is spanned by the union of the above PAOs and all SOMOs. In other words, the SOMOs have a dual role: they are occupied in the spin-up and unoccupied in the spin-down orbital set.

### 3.4 Pair Energy Calculation

The approximate MP2 pair correlation energies are evaluated for each LMO using nearby virtual orbitals residing in the PDs of the LMOs. The PDs are built using our modified^38,89^ Boughton–Pulay (BP) domain construction scheme^111^ similar to our closed-shell implementations. Briefly, the BP algorithm selects a compact list of atoms and corresponding AOs so that the overlap of the projection of the input MO onto the selected AOs with the input MO will be larger than the specified threshold (*T*). In other words, the MOs projected onto their BP AO list exhibit a well-controlled truncation error of 1 – *T*.^38^ For the PD construction, the BP atom lists are obtained for each LMO and PAO with completeness criteria of *T*_PDo_ = 0.999 and *T*_PDv_ = 0.98, respectively. The SOMOs are part of both the occupied and virtual subspaces; thus, both of these BP atom lists are assembled for them.

The PAO list of the PD contains the PAOs derived from the AOs in the BP domain of the PD’s LMO. Additionally, if a BP list of a SOMO obtained with the *T*_PDv_ criterion overlaps with the BP list of the LMO, then this SOMO is appended to the spin-down PAO list of the PD. The atom and AO lists of the PD are obtained as the union of the BP lists of all MOs (LMO, PAOs, SOMOs) of the PD. The MOs of the PD are projected onto the AO basis of the PD, and the PAOs (and potential spin-down SOMOs) are orthogonalized within this truncated AO basis,^38^ leading to different spin-up and spin-down MOs. Then, for the noniterative evaluation of the MP2 pair energies, the PD’s virtual space is canonicalized separately for the spin-up and spin-down MOs. Finally, the multipole approximated opposite-spin MP2 pair correlation energies^38^ are evaluated as
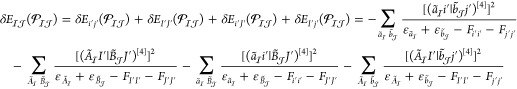
7Here, subscript  or  of the virtual orbitals indicates that the virtual indices run over the virtual subspace of the PD of the corresponding LMO. Furthermore,  denotes the pseudocanonical orbital energy of orbital , and *F*_*i*′*i*′_ (*F*_*I*′*I*′_) is the diagonal element of the spin-up (spin-down) Fock matrix. The ERIs of  written in the Mulliken notation are obtained using the multipole expansion up to the fourth order, that is, including terms with dipole–dipole, dipole–quadrupole, quadrupole–quadrupole, and dipole–octopole moments.^38^

With that, the LMO pair of  and  is classified as a strong pair if ; otherwise, the pair is treated as a distant pair, and its contribution is added to the final MP2 correlation energy (see eq 5). Here, ε_w_ is the same strong pair threshold employed in our methods previously,^38,88−90^ and *f*_w_ is a scaling factor introduced for the following reasons. Let us consider the case when one LMO of the pair, say , is SO. Then, the second and third terms of eq 7 vanish, and therefore, all such pair correlation energies contain only half as many terms compared to the pair energy of two DO LMOs. Furthermore, if both  and  are SO, then only the first term of eq 7 survives, leading to 4 times less terms contributing to an SO–SO pair than to a DO–DO pair. In accord with this consideration, on average, we find the SO–DO (SO–SO) pair correlation energies to be twice (four times) as small as those of DO–DO pairs. To handle the strong/distant pair characterization of all pair types on an equal footing, *f*_w_ factors of 1, (1/2), and (1/4) are employed for the DO–DO, DO–SO, and SO–SO pairs, respectively. The numerical properties of this scaling are analyzed in Section 5.1. We note that a similar scaling factor of (1/3) is introduced in ref (63) in the DLPNO context for pairs involving at least one SOMO. On systems with unusually large numbers of SOMOs, the factor of (1/3) provided better numerical performance than 0.1 or 0.5.^63^ This could be explained by the fact that, for the systems explored in ref (63), (1/3) is the closest to the weighted average of (1/2) and (1/4) recommended here.

The closed-shell limit of this pair energy expression matches the formulae used in our closed-shell LMP2 method. However, due to the fact that the MP2 pair energy is evaluated on an unrestricted basis, the computational requirement is somewhat higher. In practice, this does not pose a computational bottleneck as the multipole-based MP2 pair energy calculation is very efficient compared to the remaining steps of the algorithm.

### 3.5 Extended Domain Construction

The main correlation energy contribution (first term on the right side of eq 5) of each LMO is evaluated in LMO-specific EDs of asymptotically constant size to ensure the linear scaling of this step. The ED construction scheme closely follows our algorithm developed for closed-shell systems;^38,89^ thus, only a brief summary is provided here focusing on the modifications required for open-shell systems.

The occupied space of the ED consists of the CMO and all of its strong pair LMOs. The atom list of the ED is the union of the BP atom lists obtained with a BP completeness criterion of *T*_EDo_ = 0.9999 for all LMOs of the ED. The AOs on these atoms form the AO basis of the ED. The LMOs are projected onto the AOs of their respective BP atom lists ensuring at most 1 – *T*_EDo_ truncation error and are then reorthogonalized. The virtual space of the ED is spanned by restricted PAOs originating from atoms of the PAO center domain (PCD) of the ED. The PCD is the union of the more compact BP atom lists of all LMOs in the ED obtained with *T*_o_ = 0.985. Since the PAOs tend to be more delocalized than the LMOs, they are projected onto the whole AO basis of the ED. Analogously to the case of the PD construction, the SO LMOs of the ED are appended to the spin-down unoccupied MOs of the ED. The specific combination of the Gram–Schmidt and Löwdin algorithms^112,113^ is employed for the orthogonalization of the virtual space of the ED analogously to our previous approach.^38,89^ Finally, pseudocanonical and hence unrestricted occupied and virtual orbitals are obtained for the iteration free MP2 energy formulae of the ED.

### 3.6 Integral Transformation in the Extended Domain

The correlation energy computation in the ED is accelerated using the density-fitting (DF) approximation.^114,115^ The required antisymmetrized two-electron integrals are denoted as
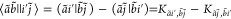
8and the **K** ERI tensors are factorized as
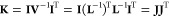
9Here, *I*_*ãi*′,*P*_ = (*ãi*′|*P*) denotes three-center two-electron integrals, and *P* refers to the auxiliary basis functions. The two-center integral matrix *V*_*PQ*_ = (*P*|*Q*) is subjected to Cholesky decomposition (**V** = **LL**^T^) yielding the **J** = **I**(**L**^–1^)^T^ tensor. We showed in ref (38) that the auxiliary basis functions residing on the atoms of the PCD can accurately expand all LMO–PAO orbital product densities of the ED; thus, the auxiliary function list of the ED is chosen accordingly.

The integral-direct ERI transformation algorithm proceeds as follows
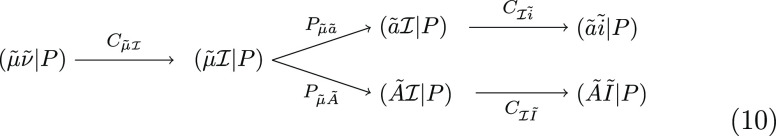
10where **C** and **P** collect the occupied and virtual MO coefficients discussed in Section 3.5. First, the (*μ̃ν̃*|*P*) AO integrals are evaluated for a shell triplet at a time using a highly optimized three-center two-electron AO integral code^116^ only for the AOs and auxiliary functions of the ED. These batches are immediately subjected to the first transformation of scheme eq 10, leading to half-transformed integrals with one index in the restricted LMO basis and then discarded. This integral-direct approach effectively makes use of the available memory and data traffic bandwidth between the lower levels of cache and the CPU. The evaluation and first transformation of the three-center ERIs are the most computationally demanding operations in our LMP2 scheme and can be performed at a similar cost as in the closed-shell implementation because restricted LMOs are employed. The introduction of this intermediate step transforming to the restricted LMO basis is thus more effective than transforming from the AO basis directly to the semicanonical occupied basis. The latter, restricted LMO to semicanonical MO transformation is performed much more efficiently as the final step of scheme eq 10. Before that, however, it is beneficial to decrease the number of operations by performing the AO-to PAO transformations (second step of scheme eq 10). Note that the number of integrals entering the second half-transformation is considerably lower than in the first step. Consequently, there is no motivation to perform the AO-to-PAO transformation in two steps by making use of the restricted PAO basis unlike in the case of the first half-transformation. In conclusion, the three-center ERIs are thus transformed to the spin-up and spin-down ED MO bases in a cost comparable to that of the closed-shell alternative.

### 3.7 Energy Contribution in the Extended Domain

Let us first note that the MP1 amplitudes appearing in the ED’s correlation energy expressions of eqs 3 and 4 are required only for a fixed *i*′ or *I*′ index. Thus, we recommended^38,88^ circumventing the redundant evaluation of MP1 amplitudes via CD^91^ or LT^29,30^ techniques. The benefit is that, by factorizing the energy denominators, we can directly evaluate the amplitudes with mixed restricted LMO and semicanonical ED MO indices, e.g.,
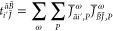
11Here, ω labels the summation index over the Cholesky vectors or integration quadrature weights used in the LT. Since the doubles amplitudes can have both spin-up and spin-down indices, it is more beneficial to obtain spin-independent Cholesky vectors or quadrature points. For instance, in the case of LT, this is achieved using the range [min(ε_*ĩ*_, ε_*Ĩ*_), max(ε_*ã*_, ε_*Ã*_)] to determine the weights (*w*_ω_) and quadrature points (*t*_ω_). Then, the **J̅** integrals of eq 11 can be constructed, e.g., as
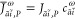
12where *c*_*ãĩ*_^ω^ denotes the elements of the Cholesky vector, or in the case of LT
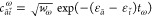
13Utilizing these integrals, the energy denominator free expression of eq 11 can be directly written down with *J̅*_*ãi*′,*P*_^ω^ obtained from *J̅*_*ãĩ*,*P*_^ω^ via the unitary transformation of the occupied MO index.

Let us note that our original closed-shell LMP2 algorithm employed an additional, so-called natural auxiliary function (NAF)^117^ approximation to compress the auxiliary function space of the EDs.^38,88^ To simplify the Ansatz, the NAF approximation is not invoked in the EDs in our most recent closed-shell LMP2 approach.^89^ NAFs are only employed in combination with natural orbitals for our LNO-CC methods.^89,90^ For the sake of compatibility, the open-shell extension of the NAF approach^118^ is not employed here at the MP2 level.

The remaining amplitudes with the other three spin cases (*t*_*i*′*j̃*_^*ãb̃*^, *t*_*I*′*J̃*_^*ÃB̃*^, and *t*_*I*′*j̃*_^*Ãb̃*^) are evaluated analogously using the appropriate spin cases of the *J̅* tensors. Finally, the RO-LMP2 energy contribution of orbital  can be evaluated in its ED as
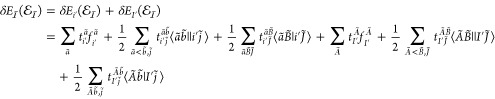
14Here, we exploit the permutational symmetries of *t*_*i*′*j̃*_^*ãb̃*^ and *t*_*I*′*J̃*_^*ÃB̃*^ in the second and fifth terms; thus, the evaluation of eq 14 takes about three times more operations than its closed-shell analogue.

### 3.8 Contribution of Single Excitations

Special attention has to be devoted also to the energy contribution of single excitations (first and fourth terms of eq 14). These contributions appear because the presented Ansatz assumes a reference determinant with unrestricted orbitals, but an ROHF/ROKS/QRO reference is employed instead of UHF. Consequently, the occupied-virtual block of the complete molecule Fock matrix written in the basis of the semicanonical MOs is nonzero even without any of the above local approximations. Additionally, the truncation of the MOs in the EDs would result in a second contribution to *f*_*i*′_^*ã*^ and *f*_*I*′_^*Ã*^ of eq 14. The reason for that is a small contamination of the projected occupied (virtual) orbitals of the ED from the virtual (occupied) subspace spanned by the untruncated MOs of the entire system. In the closed-shell context, we found this source of error small and well-controlled by the BP completeness criteria governing the truncation of the ED’s LMOs.^38^ Previously, it was found best not to include these artificial off-diagonal Fock-matrix contributions into the ED’s correlation energy contribution. However, this strategy is more challenging to follow for the open-shell case because one cannot simply discard the correlation energy contributions of the single excitations. To maintain the exact MP2 energy as the approximation-free limit of the present local scheme and to handle the off-diagonal Fock-matrix contributions comparably to the closed-shell case, the two effects are separated as follows.

Let us recognize that, if we consider the Fock matrix in the MO representation, the majority of the above nonorthogonality error would originate from its dominating diagonal elements. However, only the off-diagonal occupied-virtual block is required for the correlation energy expression. Therefore, we build the *f*_*i*′_^*ã*^ and *f*_*I*′_^*Ã*^ quantities in each ED only from the off-diagonal part of the original semicanonical Fock matrices. The latter are computed in the AO basis at the end of the complete molecule SCF computation as
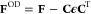
15Here, **F** and **F**^OD^ are the complete Fock matrix and its off-diagonal part in the AO basis, respectively, **C** holds the unrestricted MO coefficients, and **ϵ** is a matrix with the corresponding orbital energies on its diagonal. The benefits of storing the additional (spin-up and spin-down) **F**^**OD**^ matrices are illustrated with the example of vitamin E succinate (see Section 4.2). Using the complete **F** to compute the first and fourth terms of eq 14 would result in a 124% relative error in the singles contribution or in a 0.1% relative error with respect to the total correlation energy. Compared to that, replacing **F** by **F**^OD^ in the calculation of the *f*_*i*′_^*ã*^ and *f*_*I*′_^*Ã*^ matrices, the error in the singles contribution reduces to 0.01%, which is negligible from the perspective of the total correlation energy. For clarity, the complete Fock matrices are employed everywhere else in the algorithm, such as for the semicanonicalization of PD or ED orbitals. The use of **F**^OD^ is limited to the energy contribution of single excitations.

Let us also note that the energy contribution of single excitations is omitted from the second-order contribution of DH density functionals to ensure compatibility with the conventional implementations.^119^

### 3.9 Approximate Long-Range Spin Polarization

Here, we analyze the spin-polarization effect of the SOMOs on the MP1 amplitudes of the EDs. The present Ansatz employs unrestricted amplitudes where the contributions of spin-up and spin-down MOs to the correlation energy differ because of their different interactions with the spin-up SOMOs. The reason for that is the construction of unrestricted semicanonical MOs in the EDs even if the ED’s amplitudes are otherwise computed independent of each other, that is, they are not coupled. This spin-polarization effect takes place in all EDs, which include at least one SOMO, and thus we take this effect into account in its full extent.

The other case when the ED does not contain any SOMO is of more interest here. In these “doubly occupied MO-only” EDs, the equivalence of the spin-up and spin-down MOs originating from the restricted LMOs and PAOs of the ED is split only because their semicanonicalization in the ED is performed with the respective spin-dependent Fock matrices. In other words, in these EDs, there is no direct mixing between the SOMOs and the spin-up MOs of the doubly occupied space of the ED upon canonicalization. Note that the most important second-order contribution to the long-range and spin-polarized interaction of the SOMOs and the CMOs of such doubly occupied MO-only EDs is already taken into account via the distant pair correlation energy terms. What remains in such domains is a secondary spin-polarization effect caused by the interaction with the SOMOs through the spin-dependent Fock matrices resulting in the splitting of the orbital energies of the ED’s MOs. However, when such CMOs are distant pairs with all SOMOs, we expect that the magnitude of this long-range effect decreases rapidly.

An option has been implemented to exploit the long-range decay of spin polarization. When this is activated, the EDs without any SOMOs are treated as closed-shell subsystems, and their LMP2 energy contributions are calculated using the closed-shell formulae. This requires the introduction of an approximation: in these doubly occupied MO-only domains, the canonicalization step is performed with the average of the spin-up and spin-down Fock matrices projected onto the ED. We note in passing that alternatively, the MP1 wave function could also be spin-adapted, leading to a different Ansatz,^65^ but the applicability thereof in combination with the present local CD/LT techniques is yet to be explored.

The benefit of the introduced approximation is that the ED’s canonical MOs remain restricted, and the complete ED computation can be performed using the closed-shell algorithm. Consequently, the memory requirements of such EDs can be cut in half, and the operation count needed for the doubles amplitude evaluation can be decreased by about a factor of three. Moreover, our numerical experience presented in Section 5.4 shows that this long-range spin-polarization effect can indeed be approximated with negligible loss of accuracy.

### 3.10 Scaling of the Algorithm

The computational requirement of the presented open-shell approach is only moderately higher than that of the analogous closed-shell one, achieving for the rate-determining steps asymptotically linear scaling with system size.^38^ To verify this, the runtimes of the fifth-power scaling DF-MP2 and the present LMP2 methods were measured for quasi-linear [Th-(CH_2_)_n_-Th]^2+^ diradicals, where Th denotes thiophene rings attached to the end of the alkane chains.^120^ Detailed timing data are presented in Section S1 of the Supporting Information. In these measurements, canonical DF-MP2 exhibited an -scaling, which is somewhat lower than its formal -scaling. This can be understood as the most time-consuming step is still the -scaling integral transformation even for the largest chain. In comparison, the LMP2 algorithm exhibits clear linear scaling, which sets in already for the smallest systems. Because of the redundancy-free evaluation of the LMP2 amplitudes, the DF-MP2 and LMP2 calculations take comparable time only up to about 50 atoms followed by the clearly superior performance of LMP2 for larger systems.

For the sake of completeness, we note that the PAO construction and the multipole-based pair energy evaluation currently scale with the third and second powers of the system size, respectively, but constitute only a few percent of the total runtime even for our largest examples. The same observation can be made for the cubic-scaling localization of the occupied orbitals. The most computationally demanding part of an open-shell LMP2 calculation is thus the SCF calculation, which can also be accelerated to cubic scaling via local approximations as discussed in Section 3.1.

The memory requirement of the algorithm is even closer to its closed-shell analogue. The open-shell LMP2 program requires the storage of six matrices with dimensions equal to the number of basis functions. In comparison, the preceding SCF procedure needs eight such matrices. Moreover, all arrays related to the EDs are asymptotically constant in size, and thus the memory requirement of the open-shell LMP2 algorithm is again lower than that of the preceding SCF calculation, just as for our closed-shell implementation.

## 4 Computational Details and Test Systems

### 4.1 Technicalities

The presented RO-LMP2 approach is implemented in the Mrcc suite of quantum chemical programs^121,122^ and will be made available in a forthcoming release of the package. The default or Normal threshold values controlling the accuracy of the local approximations are collected in Table S1 of the Supporting Information. These settings correspond to the Normal threshold combination employed currently in the closed-shell LMP2 approach,^89^ which are the tighter settings introduced in ref (38).

The performed calculations utilize the split valence and triple-ζ valence basis sets including polarization functions (def2-SVP and def2-TZVP) developed by Weigend and Ahlrichs,^123^ Dunning’s (augmented) correlation-consistent polarized valence basis sets [(aug-)cc-pVXZ, X = D, T, and Q],^124^ and for third-row atoms, the revised (aug-)cc-pV(X + d)Z basis sets^125^ were also employed. The corresponding auxiliary basis sets of Weigend et al. were used for all AO bases.^126^ Extrapolations toward the complete basis set (CBS) limit were performed according to standard formulae for both the HF^127^ and correlation energies.^128^

The DF approximation was employed in all HF and reference canonical MP2 calculations. The evaluation of the exchange contribution in the HF calculations was accelerated by utilizing local fitting domains as implemented in the Mrcc package (see Section 3.1) for systems containing more than 500 atoms. The Boys localization^109^ scheme was chosen for the construction of the LMOs in each presented LMP2 calculation. The core electrons, including the subvalence electrons for the iron and cobalt atoms, were kept frozen in the correlation calculations. The energy denominators of the EDs were factorized via Cholesky decomposition,^91^ with an automatically determined number of Cholesky vectors such that the diagonal elements of the residual matrix were less than 10^–4^.

The statistical measures utilized for accuracy characterization are the maximum absolute error (MAX), mean absolute error (MAE), and the standard deviation of the absolute error (STD), the latter measuring the consistency of the errors. Relative energy differences with respect to a reference energy (*E*_DF-MP2_^c^) are obtained as (100%)·(*E*_LMP2_^c^ – *E*_DF-MP2_^c^)/*E*_DF-MP2_^c^.

The presented wall-clock times were measured with an 8-core 3.0 GHz Intel Xeon E5-1660 and a 20-core 1.3 GHz Intel Xeon Gold 6138 CPU.

### 4.2 Benchmark Sets and Test Systems

The RO-LMP2 correlation energies are benchmarked on three test sets composed of small to medium-sized open-shell molecules with an average (maximum) system size of 11 (23) atoms. The first test set collects 30 radical stabilization energies (RSE30) and is a 30-species selection from the RSE43 compilation^129^ as defined in ref (130) and reoptimized in ref (94). Furthermore, 21 adiabatic ionization potentials of organic species (IP21) are considered for systems of ref (94). The structures of the neutral systems were taken from ref (94), while the geometries of the ions were optimized using unrestricted B3LYP with the cc-pV(T + d)Z basis (see the Supporting Information). Finally, a set of 12 singlet–triplet energy gaps of aryl carbenes^131^ (AC12) was also investigated.

**Figure 2:**
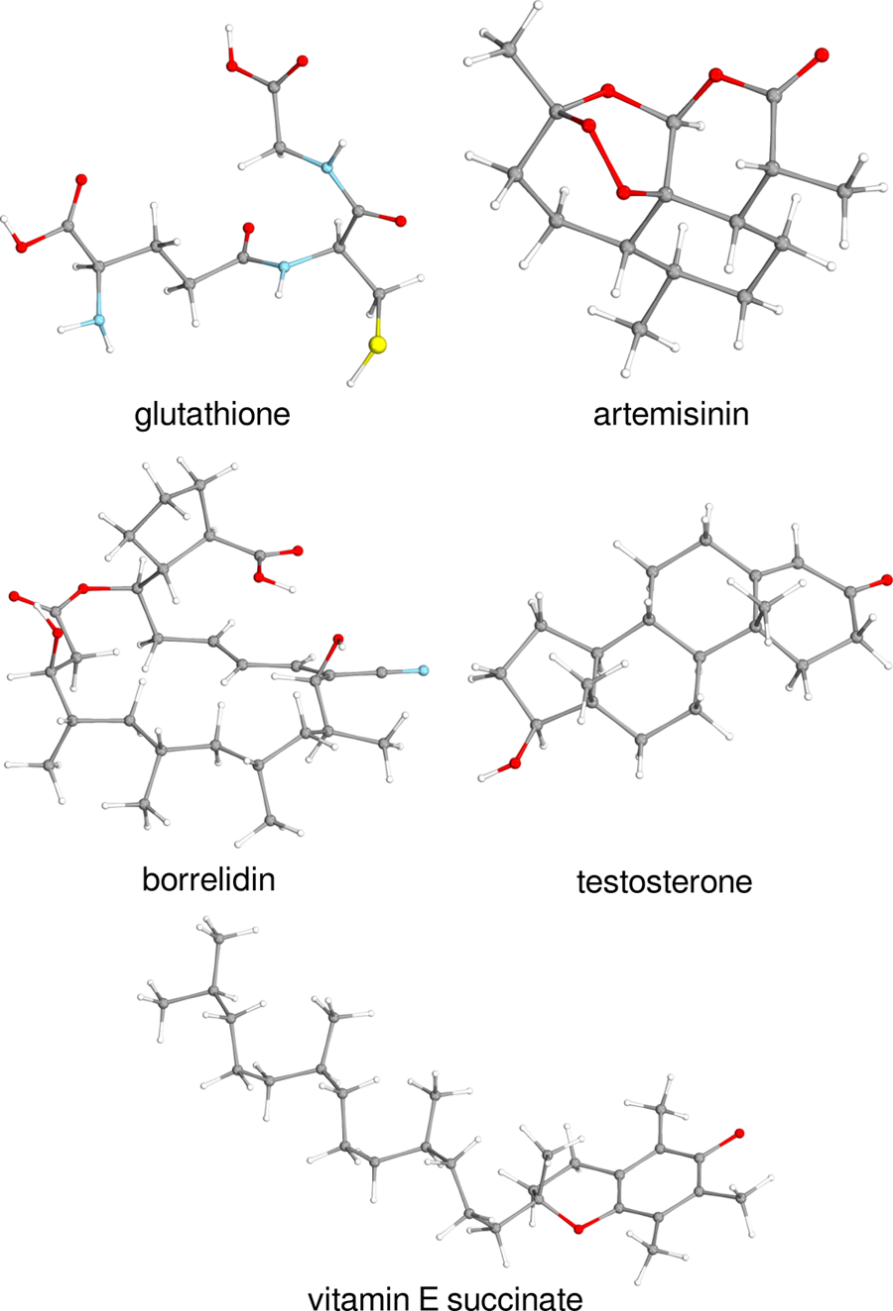
Structures of the medium-sized organic molecules studied in Sections 5 and 6.5.

Five processes involving larger open-shell systems of 42–81 atoms were also selected for the accuracy assessment. These are the radical stabilization of vitamin E succinate, the singlet–triplet energy gap of artemisinin (structures taken from ref (63)), and the vertical ionization potential of testosterone, borrelidin, and glutathione (taken from ref (94)). The corresponding structures are depicted in Figure 2.

**Figure 3:**
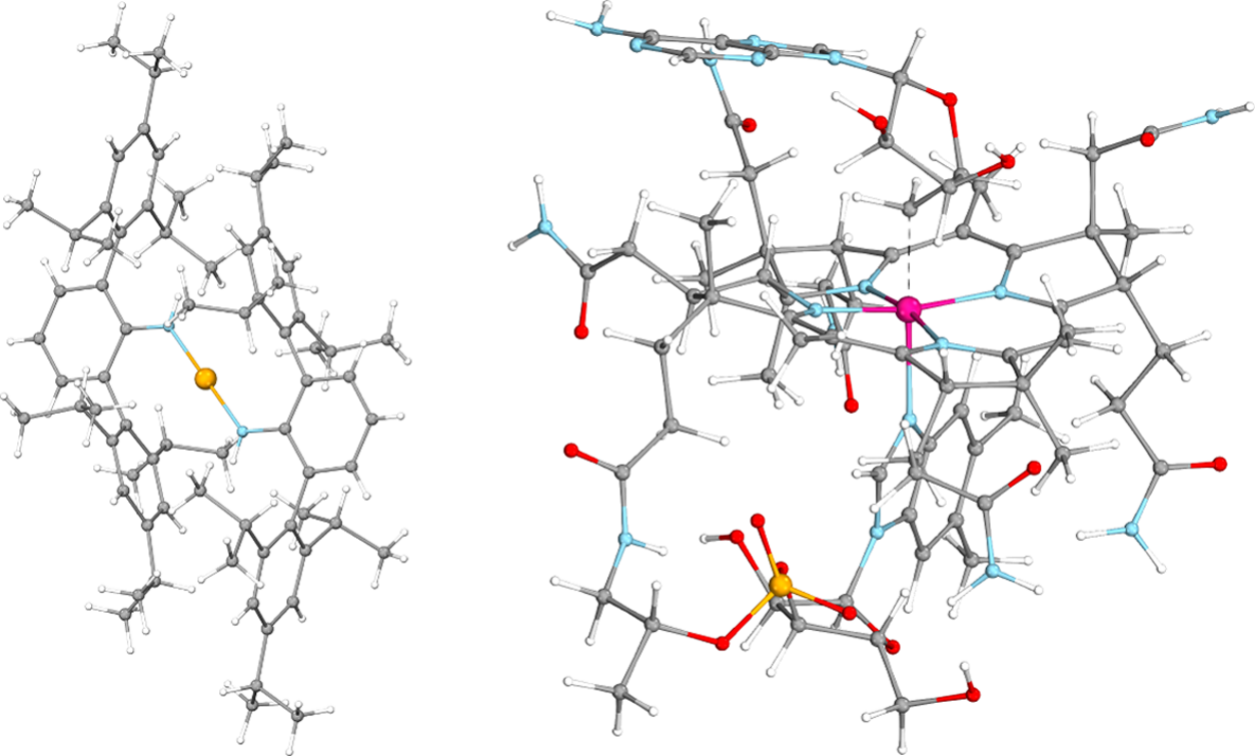
Structure of the FeC_72_N_2_H_100_ complex^120^ (on the left) containing 175 atoms, and the structure of the 5′-deoxyadenosylcobalamin (dAdoCbl) of 209 atoms (on the right). The dashed line in dAdoCbl marks the breaking Co–C bond leading to the Cbl and 5′-deoxyadenosyl radicals.^132^

Large-scale calculations were carried out for a three-dimensional iron(II) complex of 175 atoms^120^ in its quintet and triplet spin state (see Figure 3). Additionally, a homolytic bond-breaking reaction involving the coenzyme B_12_ (5′-deoxyadenosylcobalamin, dAdoCbl) with open-shell systems of up to 179 atoms [the Cob^II^alamin (Cbl) radical] was also considered^132^ (see Figure 3).

**Figure 4:**
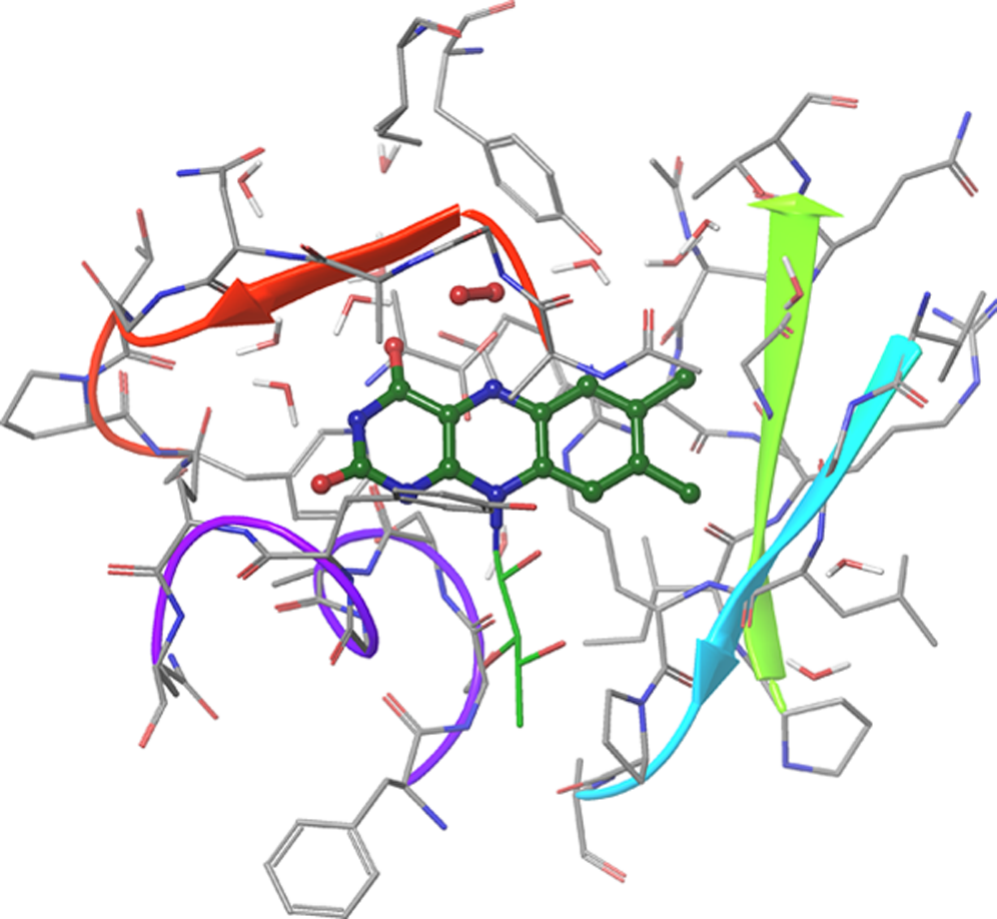
Triplet state of the d-amino acid oxidase (DAAO) model.^133^

To demonstrate the current capabilities of our LMP2 method, calculations for even larger systems were carried out for a 565-atom model of bicarbonate in photosystem II (PSII)^63^ (Figure 9) and for a 601-atom model of the d-amino acid oxidase (DAAO)^133^ (Figure 4).

**Figure 5:**
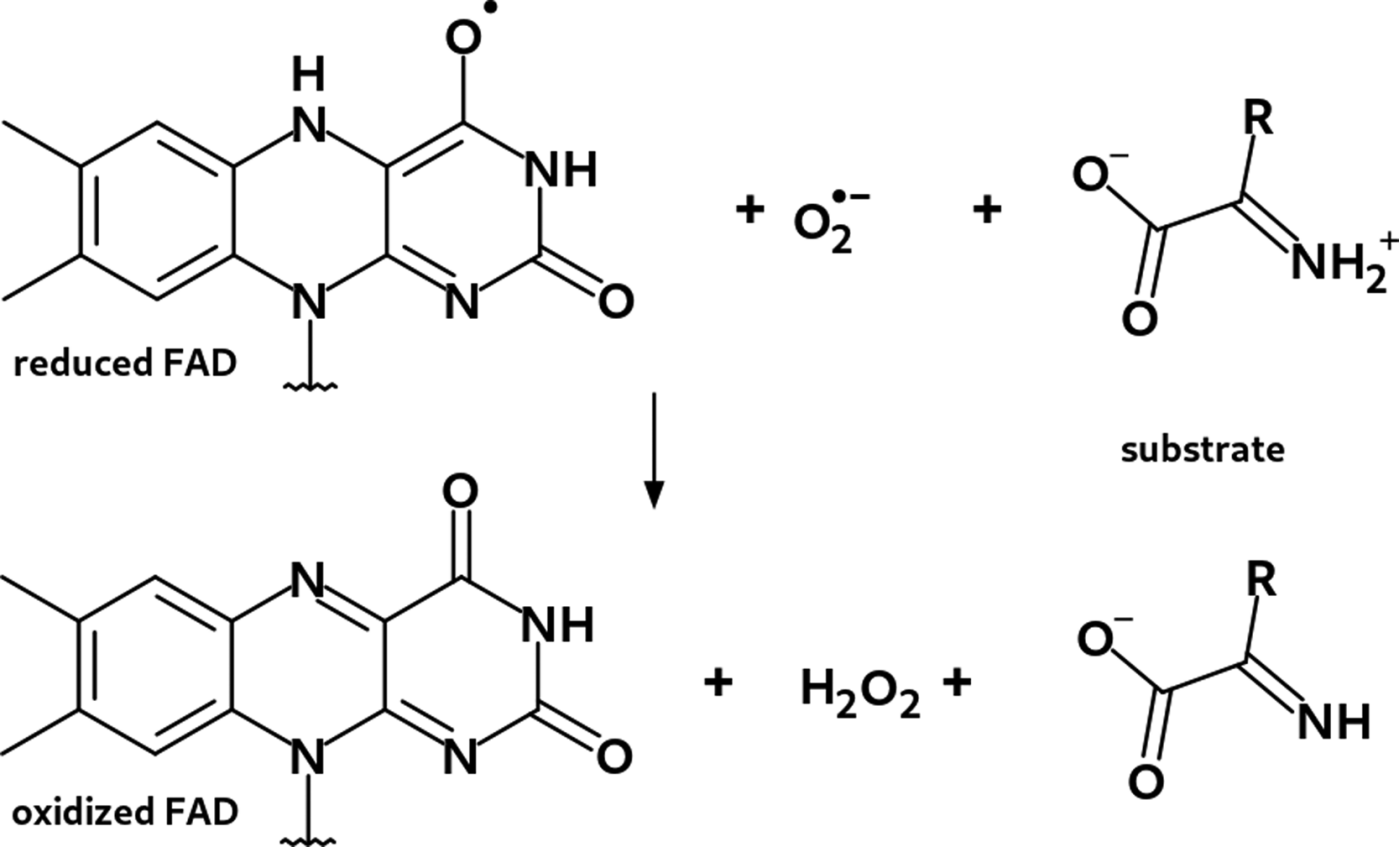
Investigated triplet reactant and singlet product states of the oxidative half-reaction catalyzed by DAAO.^133^

Following the recent mechanistic study of Kiss and Ferenczy,^133^ two steps are taken from the DAAO-catalyzed oxidation of d-alanine along the oxidative half-reaction. As illustrated in Figure 5, the reduced form of the flavin moiety of the flavin adenine dinucleotide (FAD) cofactor is reoxidized by O_2_. The diradical reactant state of Figure 5 results from a single electron transfer from reduced FAD to O_2_, leading finally to the oxidized form of FAD and H_2_O_2_. Models of the corresponding triplet and singlet states of the structures labeled by O1^T^ and O3^CSS^ in ref (133) are provided in the Supporting Information.

The bicarbonate system of PSII contains an iron(II) center for which the SCF computations were found complicated for both the quintet and triplet spin states (see Section S3 of the Supporting Information). Satisfactory UHF-based QRO references were obtained using the def2-TZVP basis set, as well as a mixed basis set labeled by def2-SVP’, which includes def2-SVP for all atoms, except for the def2-TZVP basis used for the Fe atom.

## 5 Accuracy of the Local Approximations

The truncation threshold dependence of the RO-LMP2 approach is documented in this section compared to approximation-free DF-MP2 references showing the systematic convergence of the introduced local approximations. The majority of the approximations have been extensively benchmarked in our related studies on closed-shell systems.^38,88−90^ Therefore, convergence tests illustrating individual approximations focus on the two parameters (ε_w_ and *T*_EDo_) responsible for the bulk of the local error. Open-shell-specific approximations, which did not appear before, are also thoroughly benchmarked. For the remaining truncation parameters, which affect the closed- and open-shell systems similarly, such as the BP parameters of the PDs or the order of multipole expansion, the previously assessed values are adopted.^38,89^ Note that such approximations are also active and hence tested in the benchmarks of Section 6.

### 5.1 Strong Pair Classification

As discussed in Section 3.4, the pair correlation energy expression of eq 7 does not contain an equal number of nonzero terms for orbital pairs involving different numbers of SOMOs. To handle the strong/distant pair classification of the DOMO–SOMO and SOMO–SOMO pairs on an equal footing with that of the DOMO–DOMO pairs, we propose to scale the pair energy threshold (ε_w_) by *f*_w_ factors of (1/2) and (1/4) for the pairs including one or two SOMO(s), respectively.

**Figure 6:**
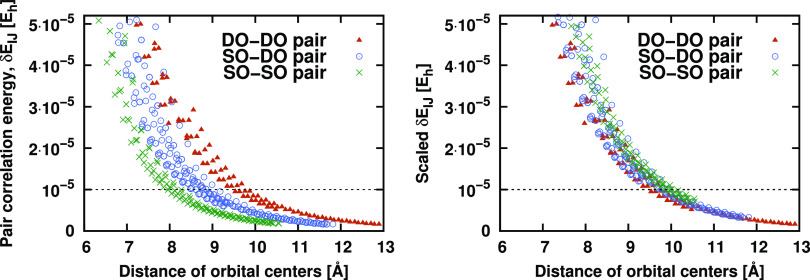
MP2 pair correlation energies () as a function of the real-space distance between the centers of the orbitals separately for DO–DO, DO–SO, and SO–SO pairs. The left panel plots unscaled  values, while  is plotted in the right panel with  for the DO–DO, DO–SO, and SO–SO pairs, respectively.

The numerical behavior of this approach is illustrated in Figure 6, which plots pair correlation energy contributions  as a function of the real-space distance between the centers of LMOs *I* and *J*. The  values are collected from multiple systems containing two methyl carbene species placed at varying distances from each other, with both methyl carbene subsystems being in their local triplet state. The left panel, collecting unscaled pair energies, illustrates that pairs involving different numbers of SOMOs gather into three distinct clusters of points. This verifies our expectation that for pairs with comparable orbital center distances, smaller pair correlation energies are obtained for SO–SO or DO–SO pairs than for DO–DO pairs. Consequently, the curves of the three groups of unscaled pair energies intersect the default pair energy threshold (dashed horizontal line) at different distances. This reveals a potential bias in the strong/distant pair classification of pairs involving SOMOs. However, our goal is to ensure comparable classification for all pairs exhibiting a similar pair distance or interaction strength regardless of their occupation. To that end, we examine the distance dependence of the same pair correlation energies scaled by , that is, by 2 and 4 for the DO–SO and SO–SO pairs, respectively. This emulates the use of the *f*_w_ ε_w_ strong pair threshold instead of ε_w_. The resulting scaled pair energies collected in the right panel of Figure 6 indeed exhibit the same trend for all three types of pairs independent of the occupation. Another beneficial consequence of using the scaled pair threshold is that the chance of including the SOMOs in the EDs increases. These SOMOs often play an important role in the chemical processes of open-shell species, and therefore, their improved description is advantageous.

### 5.2 Strong Pair Selection

Here, we assess the convergence of the LMP2 correlation energy toward the canonical DF-MP2 reference as a function of the pair energy threshold (ε_w_). To that end, LMP2 calculations are performed in which all local approximations are turned off except for the strong pair criterion of the ED construction. The approximations governed by this threshold are negligible for small systems and start to operate to a considerable extent for larger molecules. Besides the correlation energies of such extended systems (42–81 atoms), the accuracy of three different kinds of relative energies is also assessed: the vertical ionization potential (VIP) of testosterone, the radical stabilization reaction energy (RSE) of vitamin E succinate, and the singlet–triplet (S–T) gap for artemisinin. The basis set of aug-cc-pVTZ is used for all species so that the tests will be performed with a large basis set including diffuse functions sufficient for realistic applications. Diffuse AOs are more challenging to handle for local approximations, and consequently, such AOs cannot be omitted in representative convergence tests.

**Figure 7:**
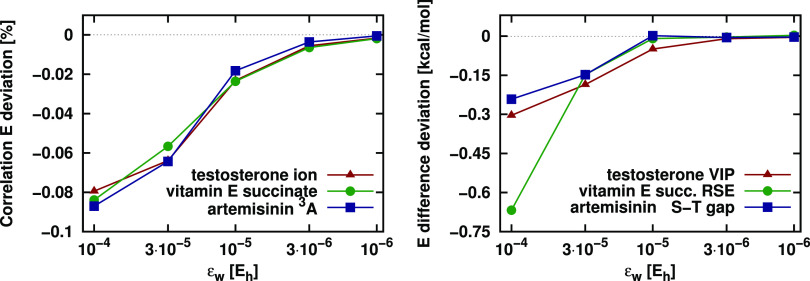
Relative LMP2 correlation energy (left) and LMP2 energy difference (right) deviations from the DF-MP2 reference for the VIP of testosterone, the RSE of vitamin E succinate, and the S–T gap of artemisinin as a function of the pair energy threshold, ε_w_.

The relative errors of LMP2 correlation energies obtained for the open-shell species (left panel) and the corresponding energy difference deviations (right panel) are depicted in Figure 7 as a function of ε_w_. Rapid convergence is observed for all cases, similar to previous findings on closed-shell systems.^38,89^ The energy differences are practically converged already at the default ε_w_ = 10^–5^*E*_h_ setting, and the largest error of 0.05 kcal/mol is negligible compared to the 217 kcal/mol VIP of testosterone. The corresponding correlation energies are also accurate up to 0.03% relative errors with this default threshold.

Note that this default value of ε_w_ = 10^–5^*E*_h_ corresponds to the tighter settings employed in ref (38), and it has been employed also as default in the context of our LNO-CC approaches^89,90^ and also with the LMP2 scheme since 2018. The strong pair selection and ED construction controlled by ε_w_= 10^–5^*E*_h_ were found to be similarly accurate previously for a number of alternative systems containing up to 260 atoms and for various reaction and interaction energies involving closed-shell systems.^38,89,90,134,135^

### 5.3 Representation of the LMOs

The second most important threshold determining the tightness of the local approximations is the BP criterion governing the completeness of the LMOs in the ED (*T*_EDo_). Together with ε_w_, these two thresholds also determine the number of atoms, AOs, and the truncation errors of the MOs in the ED.

The convergence tests for the *T*_EDo_ parameter are performed for the same open-shell species and energy differences as used in Section 5.2 for ε_w_. Again, only the local approximation corresponding to *T*_EDo_ was active, and all other approximations were turned off to separate the effect of *T*_EDo_.

**Figure 8:**
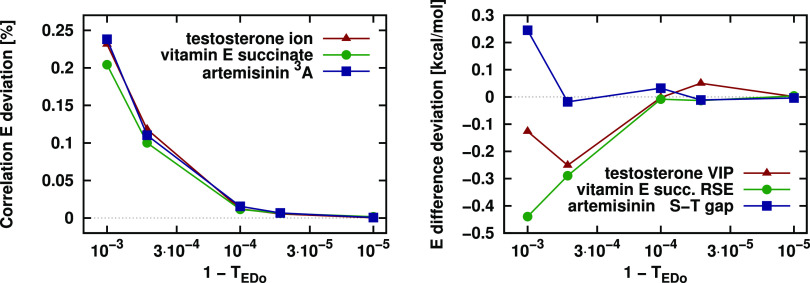
Relative LMP2 correlation energy (left) and LMP2 energy difference (right) deviations from the DF-MP2 reference for the VIP of testosterone, the RSE of vitamin E succinate, as well as the S–T gap of artemisinin as a function of the BP completeness criterion, *T*_EDo_.

The relative correlation energy (left panel) and energy difference (right panel) deviations of Figure 8 again reveal rapid convergence with increasing *T*_EDo_ toward the DF-MP2 reference. Both the correlation energies and the energy differences are converged already at *T*_EDo_ = 0.9999 (1 – *T*_EDo_ = 10^–4^ in Figure 8), which is chosen as default. We note again that this value corresponds to the tighter setting introduced in ref (38), and it is chosen as default also in our recent closed-shell LMP2 as well as LNO-CC methods.^89,90^

### 5.4 Assessment of the Long-Range Spin-Polarization Approximation

The long-range spin-polarization approximation of Section 3.9 is evaluated both on correlation energies and on energy differences with respect to LMP2 references obtained without this approximation. The approach is only active in EDs, which do not contain any SOMOs as strong pairs of the ED’s CMO. Thus, reasonably large systems have to be considered for this test to properly activate the long-range spin-polarization approximation. Accordingly, seven correlation energies and five energy differences (reaction energies, spin-state splittings, and one RSE) are benchmarked in Table 2 for systems containing 81–601 atoms. The test cases include reactions that also involve closed-shell species. For such cases, error compensation between the reactants and products cannot occur for this particular source of error because the long-range spin-polarization approximation affects only the open-shell species.

**Table 2:** Accuracy of the Long-Range Spin-Polarization Approximation Compared to Reference LMP2 Correlation Energies and Energy Differences Obtained without This Approximation^a^

					error in energy difference		
		atoms	LMOs	*E*_LMP2_^c^ error [%]	[cal/mol]	[%]	EDs without SOMOs [%]
vitamin E succinate		81	89	7.8 × 10^–7^	0.027	2.8 × 10^–4^	54
FeC_72_N_2_H_100_	^5^A	175	205	1.7 × 10^–5^	0.66	1.4 × 10^–3^	54
	^3^A		204	8.5 × 10^–6^			54
Cbl radical		179	250	7.7 × 10^–6^	0.81	1.6 × 10^–3^	68
bicarbonate	^5^A	565	789	1.2 × 10^–4^	3.5	8.7 × 10^–3^	91
	^3^A		788	1.1 × 10^–4^			92
DAAO		601	838	2.3 × 10^–7^	0.078	2.6 × 10^–4^	76

a See the text for explanation.

The last column of Table 2 collects the ratio of EDs without SOMOs, that is, the ratio of EDs affected by the approximation. Even for the smaller vitamin E succinate system, 54% of the EDs can be treated with the more efficient closed-shell formulation, while for the spin state of bicarbonate, more than 90% of the EDs are built without SOMOs. In light of the relatively large number of EDs where the approximation is activated, the relative correlation energy errors of about 10^–4^–10^–7^% for all cases are surprisingly small. This error range is comparable to or even better than that of any other employed approximation, including the DF approach. Consequently, most of the energy differences are also practically unaffected by this approximation being below 1 cal/mol for all but one example.

One should also note the key role of the SOMOs in the considered reactions, ionizations, and spin-state splittings as opposed to different possible processes occurring far from the SOMOs. This suggests that any severe approximation to the spin-polarization effects would be indicated by the investigated energy differences.

Interestingly, the quality of the approximated energy differences is similar for the systems of Table 2 even if closed-shell species are also involved (cf., vitamin E succinate RSE, the formation of dAdoCbl from the Cbl radical, and the DAAO reaction). The case of bicarbonate is somewhat an outlier in Table 2; however, the error of 3.5 cal/mol (or 0.0087%) observed in the spin-state splitting is still satisfactory, especially if considering that more than 90% of the ED contributions are approximated. It is also interesting to point out that the bicarbonate system is the only one where we had to rely on QROs due to lack of a converged ROHF reference. While the QRO approach also provides an *Ŝ*^2^ eigenfunction as the reference, the QRO reference energy, and potentially also the corresponding unrestricted Fock-matrix elements, may differ from the completely variationally optimized UHF solution more than the analogous ROHF-based quantities. Therefore, the approximation of QRO-based unrestricted Fock-matrix elements by spin-averaged ones may affect the interaction of the bicarbonate’s SOMOs with the rest of the DOMOs in a somewhat more pronounced manner.

**Figure 9:**
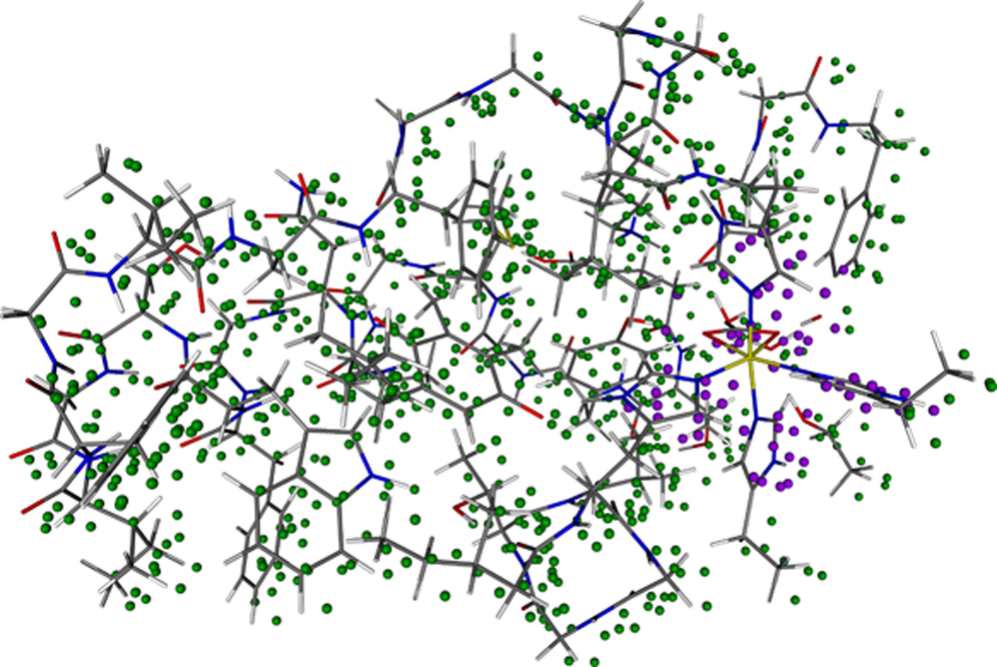
Structure of bicarbonate in PSII augmented with the spatial distribution of the centers of CMOs for all EDs. Green (purple) spheres denote ED centers with (without) active long-range spin-polarization approximation.

The spatial distribution of the EDs in which the approximation is active is visualized for the quintet state of bicarbonate in Figure 9. Green spheres denote the centers of LMOs without a strong SOMO pair (that is, without any spin-dependent interaction in their EDs), whereas purple spheres label the centers of LMOs having at least one strong pair involving a SOMO. Clearly, the EDs including at least one SOMO, in which complete open-shell treatment is required, are clustered around the Fe(II) ion, where all four SOMOs are localized. In this particular case, the SOMOs are located near the edge of the protein system; thus, the long-range spin-polarization approximation can be employed for over 90% of the EDs.

## 6 Benchmarks for Small and Medium-Sized Systems

The accuracy of RO-LMP2 correlation energies and energy differences is also benchmarked against canonical DF-MP2 references. The corresponding reference data is provided in the Supporting Information. First, statistical measures are presented for three test sets containing IPs, RSEs, and spin-state energy differences for molecules of small to medium size. Next, the accuracy is assessed also on a set of larger systems of up to 175 atoms to explore the behavior of the employed approximations with increasing system size.

### 6.1 Accuracy of Correlation Energies

The accuracy of the open-shell LMP2 correlation energies using the default settings were benchmarked on the RSE30, IP21, and AC12 test sets, containing 128 species of up to 23 atoms, thereby allowing for the statistical analysis of the correlation energies compared to the approximation-free DF-MP2 reference.

The accuracies of the LMP2 correlation energies for the RSE30, IP21, and AC12 compilations are highly satisfactory (see Tables 3–5, respectively). The relative deviations in the correlation energies (third column of these tables) are in all cases below 0.05% and are lower than 0.02% for all species in the RSE30 and IP21 sets using basis sets of various qualities as well as CBS extrapolation. The corresponding MAEs of at most 0.004% for the RSE30 and IP21 and the MAE of 0.03% for the AC12 set are also excellent. The largest errors are found for the AC12 test set with the cc-pVDZ basis set in accordance with the observation that the employed local approximations perform best for sufficiently flexible, at least triple-ζ-quality basis sets.^90^ Furthermore, these somewhat larger deviations in the correlation energies are similar for both the triplet and singlet states of the AC12 set, leading to highly accurate singlet–triplet energy gaps (see Section 6.4). The observed STD values being comparable to or even smaller than the MAE measures for all three test sets also indicate well-balanced correlation energy errors, which is beneficial for reliable energy differences. We also find that the CBS-extrapolated LMP2 energies of Table 3 maintain the accuracy of the LMP2 energies obtained with triple- and quadruple-ζ basis sets similar to our experience with closed-shell systems.^90^

**Table 3:** Relative Correlation Energy Deviations and Absolute Errors of the LMP2 Reaction Energies for Radical Stabilization Energies of the RSE30 Test Set Using the Default Thresholds

basis	error measure	error in *E*_LMP2_^c^ [%]	error in RSE [kcal/mol]
aug-cc-pV(T + d)Z	MAX	0.014	0.041
	MAE	0.003	0.010
	STD	0.003	0.011
aug-cc-pV(Q + d)Z	MAX	0.016	0.065
	MAE	0.003	0.029
	STD	0.004	0.012
CBS(T,Q)	MAX	0.017	0.097
	MAE	0.004	0.055
	STD	0.005	0.020

**Table 4:** Relative Deviations of the LMP2 Correlation Energies and Absolute Errors of the Corresponding Ionization Potentials for the IP21 Test Set Using the Default Thresholds

basis	error measure	error in *E*_LMP2_^c^ [%]	error in IP [meV]
aug-cc-pV(T + d)Z	MAX	0.016	2.03
	MAE	0.004	0.47
	STD	0.005	0.60

**Table 5:** Relative Deviations of the LMP2 Correlation Energies and Errors of the Corresponding Singlet–Triplet (S–T) Gaps for the AC12 Test Set Obtained with Default Threshold Settings

basis	error measure	error in *E*_LMP2_^c^ [%]	error in S–T gap [kcal/mol]
cc-pVDZ	MAX	0.04	0.13
	MAE	0.03	0.06
	STD	0.01	0.04
cc-pVTZ	MAX	0.04	0.13
	MAE	0.02	0.05
	STD	0.01	0.04

The relative LMP2 correlation energy deviations are collected in Table 6 for larger systems of 37–175 atoms. The relative deviation remains around the 0.05% mark for almost all entries of Table 6, matching the largest errors obtained for the smaller and simpler systems. The maximum error of 0.11% is obtained for both the quintet and triplet states of the largest FeC_72_N_2_H_100_ complex, but again this consistency leads to a negligible error in the spin-state splitting. Considering that the average system size increases by about 10 times when stepping from smaller to larger systems, the size dependence of the relative accuracy also appears excellent well above the size range where all approximations start to operate to their full extent.

**Table 6:** Accuracy of the LMP2 Correlation Energies and Energy Differences for Medium to Large Systems^a^

molecule		atoms	no. of AOs	*E*_LMP2_^c^ error [%]	Δ*E* error [kcal/mol]	time^b^ [min]
glutathione ion		37	1320	0.05	–0.05	21
artemisinin ^3^A		42	1426	0.04	–0.05	70
testosterone ion		49	1610	0.05	–0.01	77
borrelidin ion		78	2599	0.08	0.12	256
vitamin E succinate		81	2553	0.05	0.01	89
[Th–(CH_2_)_50_–Th]^2+^		166	2508^c^	0.05		5
FeC_72_N_2_H_100_	^5^A	175	2939^c^	0.11	0.005	180
	^3^A			0.11		186

a Unless otherwise noted, the calculations were carried out with the aug-cc-pV(T + d)Z basis set.

b Wall-clock times measured on an 8-core 3 GHz Intel Xeon E5-1660 processor.

c The def2-TZVP basis set was utilized.

All in all, the accuracy of the RO-LMP2 correlation energies closely matches that of closed-shell LMP2 correlation energies presented previously for a large number of closed-shell systems both in the smaller (<36-atom) and in the larger (up to 260-atom) size range.^38^ The benchmarks presented here and in ref (38) for the entire size range accessible for efficient DF-MP2 implementations indicate that highly reliable LMP2 correlation energies can be expected consistently for both open- and closed-shell systems.

To illustrate the accuracy along a full potential energy surface (PES), an example was adopted from ref (38), where the rotational barrier of ethane-1,2-diphenyl was studied using our closed-shell LMP2 approach (see Figure 2 of the Supporting Information). Here, a single hydrogen atom is removed from one of the phenyl rings to make the comparison to our previous test feasible (see Section S5 of the Supporting Information for more details). Structures at the two edges of the PES differ significantly: the phenyl groups interact weakly in the trans conformation but exhibit stronger π–π interaction in the cis arrangement. In agreement with our experience on the closed-shell analogue, we find the deviations with respect to the exact ROMP2 reference on the PES comparable to the error established above. Explicitly, the relative error varies in the narrow range of 0.014–0.029% across the PES with an MAE of 0.021%.

### 6.2 Radical Stabilization Energies

The radical stabilization reactions investigated in this section are taken from the RSE30 compilation^94^ and can be written as
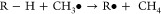
16where R^•^ denotes various radicals containing C, N, O, F, P, and S atoms. The MAEs of the LMP2 RSEs collected in Table 3 are below 0.03 kcal/mol for the aug-cc-pV(X + d)Z basis set with both X = T and X = Q, while the CBS extrapolation slightly increases the MAE to 0.05 kcal/mol. The corresponding MAX errors of 0.04, 0.06, and 0.10 kcal/mol at the triple-ζ, quadruple-ζ, and CBS(T,Q) levels, respectively, are still well within the intrinsic accuracy of MP2. The STD values of 0.01–0.02 kcal/mol underline the reproducibility of the excellent accuracy. One can also compare the accuracy of the present LMP2 results to those obtained with PNO-ROMP2 in ref (65) for the same structures and with the same aug-cc-pV(T + d)Z basis set. The two approaches perform similarly well; in terms of the MAX and MAE measures compared to the respective references, LMP2 is somewhat more accurate than PNO-ROMP2 and slightly worse than the explicitly correlated PNO-ROMP2 variant.

For this test set, the SCS-LMP2 energies were also assessed (see Table S4 of the Supporting Information) to demonstrate that the accuracy of the local approximations is consistent also for spin-scaled MP2 methods. As expected, the accuracy of both the SCS-LMP2 correlation and reaction energies matches that of LMP2; in fact, the SCS-LMP2 results are slightly but consistently better. The same trend was also observed for closed-shell systems^38^ and can be understood from the theoretical perspective because the approximations do not distinguish between the same and opposite spin terms of the SCS scheme.^11^

### 6.3 Ionization Potentials

The accuracy measures of ionization potentials are collected in Table 4. Compared to the RSE30 compilation in Table 3, both the correlation energies and the IPs obtained for the IP21 set are almost identically accurate. This excellent performance can partly be attributed to the fact that the reactants and products of the radical stabilization reactions, as well as the natural and ionized structures of the ionization processes are relatively similar and therefore some cancellation of local errors can occur. One major difference is, however, that the IPs lying in the range of about 8–14 eV (184–323 kcal/mol) are significantly larger than the RSEs. Thus, the relative deviations of the IPs compared to those of the RSEs are considerably better. Similar to the case of the RSEs, the LMP2 IP deviations are again almost twice as small as the corresponding PNO-ROMP2 errors of ref (65) with the same basis set, and LMP2 performs almost as well as the explicitly correlated PNO-ROMP2 method.^65^

### 6.4 Singlet–Triplet Energy Gaps

The energy gaps between the singlet and triplet spin states are also benchmarked for 12 aryl carbenes of 13–23 atoms taken from the AC12 compilation.^131^ Inspecting the numerical data of Table 5, the accuracy of LMP2 for S–T gaps is found similarly gratifying as for the RSEs and IPs. The MAE (MAX) measures of 0.06 (0.13) kcal/mol corresponding to the S–T gaps are again well within both the chemical accuracy and the intrinsic accuracy of MP2. It is worth noting the small improvement in accuracy observed for the more suitable cc-pVTZ basis set.

Benchmark calculations were also performed for the AC12 set using the B2PLYP^16^ functional to demonstrate the accuracy of DH density functionals approximated via our LMP2 scheme. Here, we denote the resulting method as LB2PLYP, highlighting that the second-order correlation energy contribution of B2PLYP is replaced by a corresponding LMP2 term evaluated with Kohn–Sham orbitals. Since the weight of the second-order correlation energy contribution is 0.27 for the B2PLYP functional, the accuracy of the LB2PLYP gaps is expected to be even better than that of the LMP2 gaps because the local errors are also scaled by 0.27. The numerical data of Table S9 of the Supporting Information verifies this expectation. The LB2PLYP S–T gaps are indeed found to be at least  times more accurate, exhibiting 0.01 (0.04) kcal/mol MAE (MAX) values for both basis sets. In other words, the presented local approximations operate similarly well with HF and KS orbitals in accordance with our experience for the closed-shell local DH density functional theory (DFT) variants utilizing the LMP2 method.^38^ Consequently, the LMP2 algorithm may greatly accelerate the most demanding steps in many DH density functionals with a negligible loss of accuracy.

### 6.5 Energy Differences for Larger Systems

Large-scale benchmark calculations are also presented for systems of 37–175 atoms using sufficiently large AO basis sets [aug-cc-pV(T + d)Z and def2-TZVP]. These molecules represent more faithfully the expected targets of LMP2 in practice. Furthermore, by observing potential trends in accuracy with increasing system size, one can reasonably estimate the expected deviations for even larger systems for which a reference DF-MP2 calculation becomes unfeasible. The test cases are selected so that both the pair and the domain approximations can take effect, and the domain sizes already saturate for the largest two examples.

The six energy differences collected in Table 6 include three IPs of the three ions, an RSE for vitamin E succinate, and two spin-state energy differences for artemisinin and the FeC_72_N_2_H_100_ complex. It is reassuring that none of the RSE or spin-state gap errors exceed the corresponding MAEs obtained for the same properties but with much smaller systems. Regarding the IPs, only the still highly acceptable 0.12 kcal/mol error of borrelidin exceeds the inaccuracies obtained for the IP test compilation. Thus, as expected from the underlying accurate LMP2 correlation energies, we do not find any increase in the inaccuracy of the inspected energy differences in spite of the considerable growth in system size.

Moreover, except for the Q–T gap of the FeC_72_N_2_H_100_ complex, the remaining energy differences involve both open- and closed-shell species. Consequently, the performance of LMP2 is balanced irrespective of the presence of SOMOs, allowing for the investigation of chemical processes involving both open- and closed-shell species.

Finally, representative timings are also given in the last column of Table 6 using a six-year-old, 8-core CPU. The measured runtimes of 3–4 h or less prove that RO-LMP2 is routinely applicable merely as a laptop calculation up to a few hundred atoms while maintaining the intrinsic accuracy of MP2.

## 7 Representative Applications and Computational Requirements

The capabilities and detailed computational requirements of the open-shell LMP2 algorithm are also illustrated for even larger molecules. The four systems collected in Table 7 can be arranged into two groups. The FeC_72_N_2_H_100_ complex and the Cbl radical of 175–179 atoms and of about 3000 AOs constitute the first group, as these systems are close to the capability limits of efficient open-shell DF-MP2 implementations. An additional similarity is that both systems contain a transition-metal atom, and the corresponding SOMO(s) are located close to the center of the molecule, resulting in a large number of strong pairs involving SOMO(s). The 21–25% strong pair ratio is indeed noticeably higher than the 16% obtained for the closed-shell vancomycin molecule of the same size (176 atoms) with the same settings.^38^ The corresponding EDs containing on an average (at most) about 115 (167) atoms are also significantly larger than the EDs of vancomycin built with 72 (129) atoms.

**Table 7:** Average (Maximum) Domain Sizes, Orbital Space Dimensions, DF-HF and Correlation Energies (in *E*_h_), Wall-Clock Times (in min)^a^, and Memory Requirements (in GB) for LMP2 Computations of Large Molecules

molecule	FeC_72_N_2_H_100_	Cbl radical	bicarbonate		DAAO	
atoms	175	179	565		601	
LMOs	205	250	788		837	838
SOMOs	4	1	4		0	2
AO basis	def2-TZVP	def2-TZVP	def2-SVP’	def2-TZVP	def2-TZVP	
basis functions	2939	3369	5434	10 560	11 006	
auxiliary functions	7306	8379	17 782	26 064	27 071	
strong pairs [%]	25	21	6.3	6.8	5.9	5.9
[%]	0.19	0.19	0.28	0.25	0.25	0.25
atoms in ED	114 (165)	116 (169)	138 (317)	132 (295)	124 (268)	137 (353)
AOs in ED	2086 (2854)	2342 (3278)	1376 (3195)	2634 (6015)	2449 (5408)	2693 (6943)
PAOs in ED	1020 (1812)	1017 (1828)	481 (1052)	973 (2037)	864 (1884)	902 (2930)
						
type of reference	ROHF	ROHF	QRO (UHF)		RHF	ROHF
DF-HF energy	–4156.159945	–5878.796625	–15182.8673^c^	–15197.9344^c^	–14740.9398	–14740.9040
LMP2 energy	–12.3329	–16.4723	–43.2442	–52.3847	–55.3431	–55.3319
						
HF (1 iteration)	28	43	29^b^	183^b^	152^b^	157^b^
localization	0.1	0.3	4.8	4.3	2.8	3.4
pair energies	1.2	8.7	38	4.8	11	48
integral trf.	56	157	188	451	374	639
amplitudes & *E*_LMP2_^c^	38	98	18	100	54	213
total LMP2	95	264	245	557	439	900
						
memory req.	9.8	10	4.6	17	6.7	45

a Using a 20-core 1.3 GHz Intel Xeon Gold 6138 CPU.

b Using the default local fitting domain size. The final iteration with larger fitting domains took about 3.5–4.8 times longer.

c DF-HF energies calculated with semicanonical QRO orbitals.

Considering the wall-time measurements, it is reassuring that the complete LMP2 calculation took less than the time required for three to six HF iterations; thus, the LMP2 correlation energy computation is clearly not the bottleneck in these cases. It is worth noting that compared to DF-ROHF and LMP2, the formally cubic-scaling orbital localization takes negligible time even for the largest systems of Table 7. The nonlinear-scaling steps of the LMP2 computation (see the “pair energies” line of Table 7 measuring the time of the PAO construction and the pair energy computation) are similarly efficient. As expected from the measurements performed for the closed-shell algorithm,^38^ the integral transformation and the amplitude evaluation steps dominate the time requirement of RO-LMP2 too. While the operation count required for the former is comparable to that of the closed-shell algorithm because of the use of restricted intermediate bases, the relative cost of the latter is somewhat higher for the open-shell case.

The bicarbonate and DAAO species represent the second group of examples in Table 7 consisting of 565 and 601 atoms and 10–11 thousand AOs with the def2-TZVP basis set. To the best of our knowledge, these are currently the largest three-dimensional open-shell systems for which correlated quantum chemical computations have ever been presented, at least on a single CPU. The 6–7% strong pair ratio obtained for both systems appears to be representative for protein systems of a similar size (cf. the 6% strong pair ratio for the crambin protein of 644 atoms^89^). While the average (maximum) ED sizes of bicarbonate and the closed-shell DAAO species in columns 4–6 of Table 7 are similar to or only slightly larger than the EDs of crambin holding 128 (270) atoms, the largest domain of the triplet DAAO system reaches an unprecedented size of 353 atoms. A closer inspection reveals that the CMO of this ED is a SO LMO, which expands over the entire flavin moiety close to the center of the protein. In comparison, the closed-shell singlet DAAO system has well-localized MOs and at most 268 atoms in its largest domain.

The fact that the ED computation with 353 atoms and almost 7000 AO takes only about 40 min highlights the importance and efficiency of the elaborate local approximations employed within each ED. Without exploiting the locality of the LMOs, local DF domains, the restriction of the external space to ED PAOs, the redundancy-free MP1 amplitude computation, etc., it would not be possible to compute the correlation energy contribution of several EDs reaching over 300 atoms and 6000 AOs. However, as a consequence of the delocalized SO LMO, the open-shell calculation took twice as long as its closed-shell counterpart for the analogous singlet DAAO structure of the same size. In this case, the time of integral transformation for the triplet species is longer because of the larger EDs, which would be even worse without the efficiency provided by the restricted intermediate basis.

In parallel with our experience with the closed-shell LMP2 scheme,^38,89^ the relative cost of the integral transformation compared to that of the amplitude evaluation increases with system size. Since the operation-count requirement of the integral transformation is expected to be similar for open- and closed-shell systems with comparable domain sizes, we anticipate that the SCF iterations remain the bottleneck also for RO-LMP2. It is important to be aware of the potential cost increase with highly delocalized SO LMOs, but we think that most of the practical applications will behave considerably better in this respect than in the challenging case of DAAO with its LMO spreading over the entire flavin moiety.

All in all, the RO-LMP2 computations of the largest systems required only about the time of three to six HF iterations, even if local DF is used to accelerate the HF step, and thus LMP2 is not rate-determining. Unfortunately, for such open-shell systems, the SCF procedure could take a considerably higher number of iterations than for closed-shell molecules, especially if transition-metal atoms are also involved. In many cases, one has to explore a number of options including ROHF, UHF, various density functionals, basis sets, SCF algorithms, and convergence accelerators to find a qualitatively satisfactory SCF solution. In the present study, the optimization of the quintet and especially the triplet state of the bicarbonate proved to be particularly challenging. All of our attempts for the two states accumulate into several hundreds of SCF iterations. In comparison, the triplet ROHF computation of DAAO can be considered relatively routine if accelerated with local DF domains.

It is also important to point out the benefits of the completely integral-direct and hence practically disk I/O-free LMP2 algorithm. The corresponding minimal memory requirements collected in the last row of Table 7 are also exceptionally low, being in the range of 10–20 GB for all cases except for the 45 GB allocation needed for the largest DAAO calculation. The minimal memory needed for our SCF program with local DF is also at most about 10 GB for the systems considered, but it is always beneficial to allow more memory to speed up the SCF iteration. Consequently, at least for systems accessible by current HF implementations, we do not foresee severe data bottlenecks up to the LMP2 level.

Finally, the LMP2 energy differences of the four largest examples are collected in Table 8. The quintet–triplet gap of 2.030 eV obtained for the FeC_72_N_2_H_100_ complex with both RO-LMP2 and the corresponding DF-MP2 reference is in good agreement with the 2.018, 1.852, and 2.120 eV values reported with PNO-RMP2,^65^ NEVPT2,^120^ and CASPT2,^136^ respectively. It is interesting to realize that the LMP2/def2-TZVP value of 1.759 eV obtained for the quintet–triplet gap of bicarbonate is considerably lower because of the markedly different ligand field of its Fe(II) center. While the slow basis set convergence issue of electron correlation calculations is well-known, the insufficient level of AO basis completeness provided by double-ζ-quality basis sets should still be pointed out as frequently as possible.

**Table 8:** DF-HF and LMP2 Reaction Energies and Spin-State Gaps in kcal/mol for the Four Largest Representative Examples

	def2-SVP			def2-TZVP		
	HF	Δ*E*_LMP2_^c^	Δ*E*_LMP2_^total^	HF	Δ*E*_LMP2_^c^	Δ*E*_LMP2_^total^
FeC_72_N_2_H_100_^5^A–^3^A gap	57.52	–8.92	48.60	57.56	–10.73	46.82
bicarbonate ^5^A–^3^A gap	52.62	–12.35	40.26	52.67	–12.11	40.56
Cbl + Ado → dAdoCbl	–43.38	99.84	56.46	–50.41	102.13	51.73
DAAO	20.46	8.71	29.17	22.44	7.08	29.52

## 8 Summary and Conclusions

A high-spin open-shell local MP2 (RO-LMP2) method is presented using restricted open-shell Hartree–Fock (ROHF) or Kohn–Sham (ROKS) reference determinants. The efficiency of the open-shell LMP2 approach matches that of our previous closed-shell LMP2 algorithm^38,89^ because restricted orbitals are used for the most demanding integral transformation step. The amplitudes and correlation energy contributions are evaluated using a relatively simple, unrestricted formulation, but the corresponding computational overhead is largely mitigated by a novel approximation of long-range spin-polarization effects in the correlation energy.

For closed-shell systems, the present method is identical to our closed-shell LMP2 approach. The RO-LMP2 algorithm is also especially operation-count and memory-efficient, integral-direct, OpenMP-parallel, and requires negligible hard disk use. Spatial symmetry, checkpointing, and near-linear-dependent basis sets can also be utilized.^89,90^ Usually, the entire RO-LMP2 computation takes the time of about three to six ROHF iterations; thus, even if accelerated with local approximations,^38,107,108^ the SCF optimization remains the main bottleneck, especially for large systems and/or with transition-metal atoms.

The errors caused by the local approximations are mostly below 0.1 kcal/mol and thus negligible compared to the intrinsic accuracy of MP2 as demonstrated for reactions of radicals, spin-state energy gaps, and ionization potentials. The accuracy of local spin-scaled MP2 variants is similarly excellent, while even better performance is found for double-hybrid (DH) functionals because their second-order energy contribution is usually downscaled. As an additional use case, local MP2-based corrections are often suggested to decrease the basis set incompleteness of (local) CC methods, such as local CCSD(T).^90,135,137^ The RO-LMP2 algorithm also provides important components to our high-spin open-shell LNO-CCSD(T) and higher-order LNO-CC implementations, which are currently under extensive benchmarking.

The capabilities of the RO-LMP2 implementation are illustrated on three-dimensional protein models containing up to 601 atoms and 11 000 atomic orbitals with triple-ζ basis sets. The quintet–triplet gap in the bicarbonate protein of photosystem II^63^ is relatively complicated because of the nontrivial electronic structure around the Fe(II) ion in the triplet state. The second large-scale example involving the reduction of O_2_ via d-amino acid oxidase is also challenging because of a poorly localized SOMO spreading over an entire flavin moiety. We anticipate that the common target applications of RO-LMP2 will be significantly simpler. However, it is satisfactory that such complicated systems have also become routinely available, especially if the single-node (20-core) RO-LMP2 runtimes of 9–15 h are considered. Consequently, the presented local approximations extend the reach of open-shell MP2 as well as of spin-scaled MP2 and DH DFT methods to systems of 500–600 atoms with reasonable basis sets. Except for potential bottlenecks in the ROHF/ROKS optimization, RO-LMP2 should also be applicable for even larger molecules approaching the limit of our closed-shell LMP2 and LNO-CCSD(T) codes, which is currently about 1000–2000 atoms and 45 000 atomic orbitals.^89,90^

## Supporting Information Available

Linear-scaling time measurements, list of default settings for the local approximations, PES of the ethane-1,2-diphenyl radical, reference energies for the RSE30, IP21, and AC12 test sets and the larger molecules considered as well as the accuracy assessment of the SCS-LMP2 and LB2PLYP methods; structures of the IP21 ions and DAAO models are also provided (PDF)
