# Supplementary material for: Linear-Scaling Open-Shell MP2 Approach: Algorithm, Benchmarks, and Large-Scale Applications
Source: J Chem Theory Comput. 2021 Apr 5;17(5):2886–905. doi: 10.1021/acs.jctc.1c00093 (PMC8154337; doi:10.1021/acs.jctc.1c00093)
Supplement: Supplementary file 1 — ct1c00093_si_001.pdf [file ct1c00093_si_001.pdf]

Supporting information for
"A linear scaling open-shell MP2 approach: algorithm, benchmarks, and large-scale applications"

P. Bernát Szabó, József Csóka, Mihály Kállay,* and Péter R. Nagy*

Department of Physical Chemistry and Materials Science, Budapest University of Technology and Economics, H-1521 Budapest, P.O.Box 91, Hungary

E-mail: kallay@mail.bme.hu; nagyrpeter@mail.bme.hu

S1 Linear scaling of the algorithm

The linear scaling of the measured wall-clock times is shown in Figure S1 for linear alkane chains with a positively charged thiophene ring attached to them at both ends. The structures of these systems were taken from the work of Guo *et al.*[1] For comparison, the wall-clock times of DF-MP2 are also shown. The timings were measured on a 6-core 3.5 GHz Intel Xeon E5-1650 processor.

*To whom correspondence should be addressed

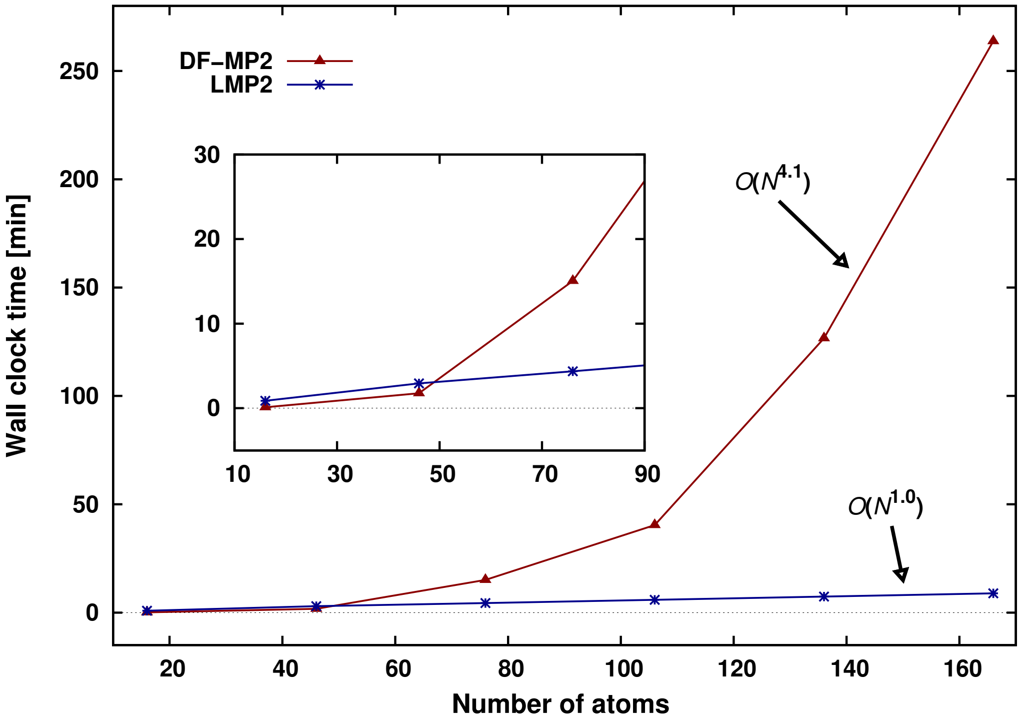

Figure S1: Wall-clock times and corresponding fitted polynomial scaling exponents of canonical DF-MP2 and LMP2 for a set of quasi-linear diradical chains of $[Th\text{-}(CH_2)_n\text{-}Th]^{2+}$ plotted against the number of atoms.

S2 Default threshold set used for RO-LMP2

The default values for the thresholds controlling the accuracy of the local approximations in RO-LMP2 are collected in Table S1.

Table S1: Default threshold set used for the present RO-LMP2 method.[a]

Symbol	Keyword[b]	Normal	description
T_{PDo}	`bppdo`	0.999	BP completeness of LMOs in PDs
T_{PDv}	`bppdv`	0.98	BP completeness of PAOs in PDs
ε_{w} [E_h]	`wpairtol`	10^{-5}	strong pair energy threshold
T_{EDo}	`bpedo`	0.9999	BP completeness of LMOs in EDs
T_{o}	`bpcompo`	0.985	BP completeness for PCD construction

[a] The threshold combinations can also be set by the `lcorthr` keyword of the Mrcc suite.[2,3]

[b] Name of the corresponding keywords in the Mrcc suit.

S3 Details of the computations for bicarbonate

The bicarbonate system of PSII contains an iron(II) center for which the quintet and triplet spin state were considered. Similar to the approach of Refs. 4 and 5, an unrestricted reference and the corresponding QROs are employed as we were unable to converge a suitable ROHF/ROKS solution. Upon reproduction of the UKS/def2-SVP reference of Refs. 4 and 5 for the quintet state, we observed considerable spin-contamination ($\langle S^2 \rangle = 7.03$) and QRO occupation numbers of $\ldots$, 1.99, 1.15, 1.0, 1.0, 1.0, 1.0, 1.0, 0.85, 0.01, $\ldots$ near the region of the SOMOs. However, it was feasible to obtain an UHF solution with $\langle S^2 \rangle = 6.01$ and QRO occupation numbers of $\ldots$, 1.997, 1.0, 1.0, 1.0, 1.0, 0.003, $\ldots$ with the def2-TZVP basis set. A similar, almost perfect quintet determinant can be obtained at the UHF/def2-SVP level if def2-SVP is replaced by def2-TZVP only on the Fe atom. Thus, besides def2-TZVP, we employ this mixed basis set throughout for bicarbonate labeled by def2-SVP'.

S4 Reference and correlation energies

All the presented calculations were carried out with an unrestricted density-fitting MP2 (DF-MP2) method based on restricted open-shell refrence determinants as implemented in the MRCC package.[2,3] DF was also employed to speed up all Hartree–Fock (HF) calculations.

S4.1 The RSE30 test set

The geometries of all species involved in the radical stabilization energies (RSEs) were taken from the work of Krause *et al.*[1] All calculations utilized ROHF references. The calculations were carried out with the aug-cc-pV(X+d)Z basis sets and the corresponding aug-cc-pVXZ auxiliary basis sets, where X = T or Q. The HF, DF-MP2, and spin-component scaled (SCS) MP2[6] energies obtained are collected in Table S2 and S3 for the closed- and open-shell species of the test set, respectively. Statistical measures for the deviations of the local SCS-MP2 RSEs from their reference values are shown in Table S4.

Table S2: Reference energies (in E_h) for the closed-shell species of the RSE30 test set.

Molecule	aug-cc-pV(T+d)Z			aug-cc-pV(Q+d)Z		
	HF	MP2	SCS-MP2	HF	MP2	SCS-MP2
CH_4	-40.213589	-0.20082	-0.20924	-40.216212	-0.21114	-0.22082
CH_3CF_3	-375.971175	-1.11117	-1.08774	-375.996670	-1.19074	-1.17430
CH_3CH_2Cl	-538.201123	-0.55780	-0.56052	-538.209042	-0.59355	-0.60038
CH_3CH_2F	-178.152156	-0.61947	-0.61926	-178.163812	-0.65897	-0.66263
CH_3CH_2OH	-154.145347	-0.61286	-0.61441	-154.155109	-0.64859	-0.65378
$CH_3CH{=}CH_2$	-117.117519	-0.51619	-0.52267	-117.124540	-0.54381	-0.55330
CH_3CHO	-152.977123	-0.57682	-0.57446	-152.986850	-0.61098	-0.61201
CH_3CN	-131.975221	-0.52201	-0.51872	-131.982956	-0.55096	-0.55057
CH_3CONH_2	-208.063284	-0.79075	-0.78153	-208.076488	-0.83768	-0.83297
$CH_3CONH-CH_3$	-247.103594	-0.96676	-0.95901	-247.119056	-1.02265	-1.02037
$CH_3COO-CH_3$	-266.940479	-0.98936	-0.97893	-266.957332	-1.04880	-1.04404
CH_3COOH	-227.905788	-0.81647	-0.80356	-227.920545	-0.86704	-0.85889
$CH_3CH(CH_2)_2$	-156.151893	-0.70123	-0.70578	-156.161254	-0.73806	-0.74655
CH_3F	-139.097158	-0.44423	-0.44301	-139.106621	-0.47459	-0.47626
CH_3NH_2	-95.255384	-0.41168	-0.41610	-95.261358	-0.43448	-0.44132
CH_3NH^+3	-95.618438	-0.40221	-0.40967	-95.624526	-0.42471	-0.43464
CH_3NHCH_3	-134.294521	-0.58634	-0.59228	-134.302833	-0.61817	-0.62748
CH_3NHCHO	-208.043788	-0.79044	-0.78229	-208.057024	-0.83723	-0.83360
$CH_3N(CH_3)_2$	-173.335330	-0.76498	-0.77158	-173.345972	-0.80568	-0.81657
CH_3NO_2	-243.761415	-0.88132	-0.86253	-243.777440	-0.93416	-0.92006
CH_3OCF_3	-450.854445	-1.34404	-1.31274	-450.884951	-1.43967	-1.41669
CH_3OCH_3	-154.128678	-0.60887	-0.61191	-154.138363	-0.64441	-0.65103
CH_3OCHO	-227.879960	-0.81458	-0.80346	-227.894578	-0.86489	-0.85847
CH_3COOCH_3	-266.940474	-0.98939	-0.97895	-266.957327	-1.04882	-1.04406
CH_3OH	-115.091974	-0.43688	-0.43753	-115.099483	-0.46351	-0.46683
CH_3PH_3	-381.889384	-0.33786	-0.35216	-381.895556	-0.35763	-0.37454
CH_3SH^+2	-438.069130	-0.36330	-0.37254	-438.075093	-0.38555	-0.39752
CH_3SH	-437.759611	-0.37063	-0.37636	-437.765287	-0.39354	-0.40202
CH_3SOOCH_3	-626.569739	-1.04322	-1.02792	-626.590019	-1.10981	-1.10080
CH_3SOCH_3	-551.662539	-0.79832	-0.79447	-551.676354	-0.84757	-0.84870
$CH_3C{\equiv}CH$	-115.909563	-0.48777	-0.48859	-115.916290	-0.51430	-0.51791

Table S3: Reference energies (in E_h) for the open-shell radicals of the RSE30 test set.

Radical	aug-cc-pV(T+d)Z			aug-cc-pV(Q+d)Z		
	HF	MP2	SCS-MP2	HF	MP2	SCS-MP2
$\cdot CH_3$	-39.573467	-0.16528	-0.17159	-39.575864	-0.17446	-0.18184
$\cdot CH_2CF_3$	-375.327447	-1.07626	-1.05068	-375.352851	-1.15472	-1.13593
$\cdot CH_2CH_2Cl$	-537.562916	-0.52374	-0.52422	-537.570612	-0.55850	-0.56290
$\cdot CH_2CH_2F$	-177.512586	-0.58509	-0.58266	-177.524065	-0.62353	-0.62476
$\cdot CH_2CH_2OH$	-153.506319	-0.57839	-0.57759	-153.515914	-0.61314	-0.61577
$\cdot CH_2CH{=}CH_2$	-116.488729	-0.50030	-0.50302	-116.495571	-0.52716	-0.53272
$\cdot CH_2CHO$	-152.345418	-0.54735	-0.54232	-152.354906	-0.58061	-0.57877
$\cdot CH_2CN$	-131.338765	-0.49529	-0.48899	-131.346324	-0.52322	-0.51962
$\cdot CH_2CONH_2$	-207.430080	-0.75739	-0.74592	-207.443092	-0.80332	-0.79618
$\cdot CH_2CONH{-}CH_3$	-246.470201	-0.93354	-0.92352	-246.485476	-0.98844	-0.98369
$\cdot CH_2COO{-}CH_3$	-266.307004	-0.95661	-0.94384	-266.323660	-1.01506	-1.00777
$\cdot CH_2COOH$	-227.272240	-0.78355	-0.76834	-227.286806	-0.83316	-0.82250
$\cdot CH_2CH(CH_2)_2$	-155.516485	-0.66982	-0.67173	-155.525674	-0.70569	-0.71132
$\cdot CH_2F$	-138.459596	-0.41156	-0.40694	-138.469051	-0.44074	-0.43876
$\cdot CH_2NH_2$	-94.628396	-0.38191	-0.38286	-94.634276	-0.40374	-0.40690
$\cdot CH_2NH^+3$	-94.969529	-0.36783	-0.37260	-94.975451	-0.38909	-0.39608
$\cdot CH_2NHCH_3$	-133.667317	-0.55742	-0.55993	-133.675537	-0.58836	-0.59403
$\cdot CH_2NHCHO$	-207.412928	-0.76237	-0.75093	-207.426039	-0.80821	-0.80105
$\cdot CH_2N(CH_3)_2$	-172.707643	-0.73628	-0.73965	-172.718189	-0.77615	-0.78361
$\cdot CH_2NO_2$	-243.120808	-0.85131	-0.82944	-243.136664	-0.90318	-0.88579
$\cdot CH_2OCF_3$	-450.217251	-1.31085	-1.27645	-450.247732	-1.40537	-1.37904
$\cdot CH_2OCH_3$	-153.497157	-0.57790	-0.57768	-153.506747	-0.61240	-0.61553
$\cdot CH_2OCHO$	-227.241409	-0.78383	-0.76940	-227.255987	-0.83316	-0.82320
$\cdot CH_2COOCH_3$	-266.302439	-0.95882	-0.94503	-266.319227	-1.01728	-1.00893
$\cdot CH_2OH$	-114.460915	-0.40584	-0.40306	-114.468365	-0.43139	-0.43104
$\cdot CH_2PH_3$	-381.247731	-0.30143	-0.31409	-381.253862	-0.32024	-0.33531
$\cdot CH_2SH^+2$	-437.422572	-0.32900	-0.33587	-437.428525	-0.35038	-0.35976
$\cdot CH_2SH$	-437.126436	-0.34227	-0.34476	-437.132144	-0.36448	-0.36949
$\cdot CH_2SOOCH_3$	-625.926947	-1.00843	-0.99098	-625.947201	-1.07404	-1.06266
$\cdot CH_2SOCH_3$	-551.022435	-0.76451	-0.75815	-551.036295	-0.81296	-0.81136
$\cdot CH_2C{\equiv}CH$	-115.277747	-0.46356	-0.46102	-115.284275	-0.48917	-0.48922

Table S4: Relative deviations of local SCS-MP2 correlation energies and absolute errors of the radical stabilization energies for the RSE30 test set obtained with default threshold settings.

basis	error measure	error in $E^{c}_{\mathrm{LSCS-MP2}}$ [%]	error in RSE [kcal/mol]
	MAX	0.013	0.030
aug-cc-pV(T+d)Z	MAE	0.002	0.009
	STD	0.003	0.009
	MAX	0.014	0.063
aug-cc-pV(Q+d)Z	MAE	0.003	0.033
	STD	0.003	0.012
	MAX	0.014	0.093
CBS(T,Q)	MAE	0.004	0.061
	STD	0.004	0.021

S4.2 The IP21 test set

The structures of the ions in this test set were optimized with the B3LYP density functional utilizing the cc-pV(T+d)Z and def2-QZVPP-RI-JK bases and unrestricted Kohn–Sham orbitals. The optimized structures are available in the Supporting Information as a zip archive. The geometries of the neutral species were taken from the work of Ma *et al.*[7] All single point calculations utilized ROHF references and the aug-cc-pV(T+d)Z basis in conjunction with the aug-cc-pVTZ auxiliary basis sets. The resulting DF-HF and DF-MP2 energies are collected in Table S5.

Table S5: DF-ROHF and DF-MP2 energies (in E_h) for all species of the IP21 test set using the aug-cc-pV(T+d)Z basis set.

	Molecule		Ion	
	HF	MP2	HF	MP2
Water	-76.060288	-0.26864	-75.652677	-0.20724
Ethane	-79.260253	-0.37500	-78.837385	-0.36527
Propane	-118.308357	-0.55162	-117.948428	-0.50350

Continued on the next page...

Table S5 – continued

Oxalic acid	-376.527853	-1.26537	-376.156714	-1.20728
Butane	-157.356325	-0.72859	-157.013004	-0.68229
Pentane	-196.404210	-0.90562	-196.057514	-0.86899
Benzoquinone	-379.369307	-1.39562	-379.038852	-1.32828
Propionyl chloride	-650.979191	-0.94381	-650.630188	-0.88072
Formaldehyde	-113.914146	-0.40184	-113.558597	-0.34149
Ethanol	-154.145347	-0.61286	-153.783643	-0.56889
Urea	-224.083069	-0.82499	-223.780981	-0.75790
Glycine	-282.951964	-1.02911	-282.634551	-0.98846
Alanine	-322.001295	-1.20877	-321.696917	-1.16754
Methylamine	-95.255376	-0.41168	-94.966109	-0.36259
Isobutene	-156.169752	-0.69500	-155.883493	-0.63801
Benzene	-230.781508	-0.96285	-230.483477	-0.91716
Butadiene	-154.979231	-0.65885	-154.692355	-0.61231
Hydrazine	-111.220706	-0.45152	-110.964249	-0.42077
Furan	-228.709885	-0.88240	-228.429395	-0.83626
Imidazole	-224.898091	-0.89534	-224.620359	-0.84326
Hexatriene	-231.893800	-0.98020	-231.632716	-0.94172

S4.3 The AC12 test set

The structures of both the singlet and the triplet states were taken from the work of Pantazis and co-workers.[8] Reference DF-ROHF and DF-MP2 energies obtained with the cc-pVDZ and cc-pVTZ basis sets are presented in Tables S6 and S7 for the singlet and triplet states,

respectively. In the DFT calculations, density-fitting restricted open-shell references together with the cc-pVDZ or cc-pVTZ basis sets were utilized. The reference B2PLYP results are shown in Table S8. The statistical measures for the deviations of the local B2PLYP values from their reference counterparts are presented in Table S9.

Table S6: Canonical DF-ROHF and DF-MP2 energies (in E_h) for the singlet species in the AC12 test set.

	cc-pVDZ		cc-pVTZ	
	HF	MP2	HF	MP2
phenylcarbene	-268.472396	-0.87982	-268.537507	-1.07118
(4-nitro)phenylcarbene	-471.950003	-1.42396	-472.075082	-1.74695
(4-methoxy)phenylcarbene	-382.366279	-1.20298	-382.467080	-1.47746
(4-hydroxy)phenylcarbene	-343.344129	-1.06167	-343.433691	-1.30521
(4-amino)phenylcarbene	-323.517534	-1.04776	-323.600423	-1.28159
(4-oxido)phenylcarbene	-342.799502	-1.07466	-342.892116	-1.32169
pentafluorophenylcarbene	-762.707854	-1.77475	-762.944296	-2.27253
methoxy(phenyl)methylidene	-382.408857	-1.20982	-382.509170	-1.48359
fluoro(phenyl)methylidene	-367.356217	-1.06296	-367.455153	-1.31581
chloro(phenyl)methylidene	-727.413720	-1.01751	-727.493608	-1.25959
diphenylcarbene	-498.046929	-1.66145	-498.165719	-2.01583
fluorenylidene	-496.889694	-1.63746	-497.005080	-1.98551

Table S7: Canonical DF-ROHF and DF-MP2 energies (in E_h) for the triplet species in the AC12 test set.

	cc-pVDZ		cc-pVTZ	
	HF	MP2	HF	MP2
phenylcarbene	-268.493000	-0.87064	-268.557799	-1.05956
(4-nitro)phenylcarbene	-471.977704	-1.41171	-472.102582	-1.73283
(4-methoxy)phenylcarbene	-382.379741	-1.19537	-382.479760	-1.46762
(4-hydroxy)phenylcarbene	-343.357489	-1.05413	-343.446415	-1.29554
(4-amino)phenylcarbene	-323.528054	-1.04117	-323.609865	-1.27278
(4-oxido)phenylcarbene	-342.775540	-1.06717	-342.867236	-1.31298
pentafluorophenylcarbene	-762.730695	-1.76318	-762.966511	-2.25940
methoxy(phenyl)methylidene	-382.384574	-1.19461	-382.484925	-1.46576
fluoro(phenyl)methylidene	-367.345993	-1.04937	-367.445381	-1.29900
chloro(phenyl)methylidene	-727.417500	-1.00462	-727.498080	-1.24348
diphenylcarbene	-498.064613	-1.64949	-498.183125	-2.00149
fluorenylidene	-496.904886	-1.63275	-497.019879	-1.97864

Table S8: Reference B2PLYP energies (in E_h) of the AC12 test set using a restricted open-shell Kohn–Sham formulation.

	singlet		triplet	
	cc-pVDZ	cc-pVTZ	cc-pVDZ	cc-pVTZ
phenylcarbene	-269.934259	-270.065243	-269.939848	-270.069629
(4-nitro)phenylcarbene	-474.288935	-474.517978	-474.301141	-474.529541
(4-methoxy)phenylcarbene	-384.361263	-384.554482	-384.360268	-384.551986
(4-hydroxy)phenylcarbene	-345.109190	-345.280894	-345.108129	-345.278600
(4-amino)phenylcarbene	-325.251389	-325.414374	-325.247427	-325.408836
(4-oxido)phenylcarbene	-344.578432	-344.755711	-344.543449	-344.719670
pentafluorophenylcarbene	-765.778714	-766.169038	-765.783555	-766.173349
methoxy(phenyl)methylidene	-384.407663	-384.600192	-384.368171	-384.559661
fluoro(phenyl)methylidene	-369.142609	-369.325357	-369.115714	-369.297336
chloro(phenyl)methylidene	-729.426327	-729.587084	-729.412994	-729.573355
diphenylcarbene	-500.773582	-501.012717	-500.776690	-501.014769
fluorenylidene	-499.446252	-499.812231	-499.586013	-499.816249

Table S9: Relative deviations of local B2PLYP correlation energies and absolute differences of the singlet–triplet (S–T) gaps for the AC12 test set obtained with default thresholds.

basis	error measure	error in $E^{c}_{LB2PLYP}$ [%]	error in S–T gap [kcal/mol]
cc-pVDZ	MAX	0.046	0.036
	MAE	0.025	0.009
	STD	0.012	0.011
cc-pVTZ	MAX	0.043	0.023
	MAE	0.021	0.010
	STD	0.010	0.007

S4.4 Larger systems

Table S10 collects reference DF-ROHF and DF-MP2 energies for all species appearing in Table 6 of the main text. The calculations in the last three rows of the table utilized the def2-TZVP basis set, while all other calculations employed the aug-cc-pV(T+d)Z basis set.

Table S10: Reference DF-ROHF and DF-MP2 energies (in E_h) for the species appearing in Table 6 of the main text.

species	HF	MP2
glutathione molecule	-1399.106134	-3.91061
glutathione cation	-1398.796372	-3.87480
artemisinin singlet	-955.382103	-3.77388
artemisinin triplet	-955.219760	-3.74850
testosterone molecule	-885.825041	-3.74264
testosterone cation	-885.546369	-3.67601
borrelidin molecule	-1589.488204	-6.46272
borrelidin cation	-1589.194235	-6.42348
vitamin E succinate molecule	-1277.393569	-5.576880
vitamin E succinate radical	-1276.776808	-5.533299
$[Th-(CH_2)_{50}-Th]^{2+}$	-3053.402254	-9.94094
$FeC_{72}N_2H_{100}$ triplet	-4156.068224	-12.36411
$FeC_{72}N_2H_{100}$ quintet	-4156.159945	-12.34700

S5 Potential energy surface of the ethane-1,2-diphenyl radical

The potential energy surface (PES) along the dihedral angle of the ethane moiety of the ethane-1,2-diphenyl radical is examined, to demonstrate the quality of the RO-LMP2 PESs (see Figure S2). The structure of the radical was obtained by removing a single hydrogen in the *para* position from one of the phenyl rings of ethane-1,2-diphenyl. The geometries of the system were optimized for each dihedral angle considered, with the PBE density functional using the def2-TZVP basis set.

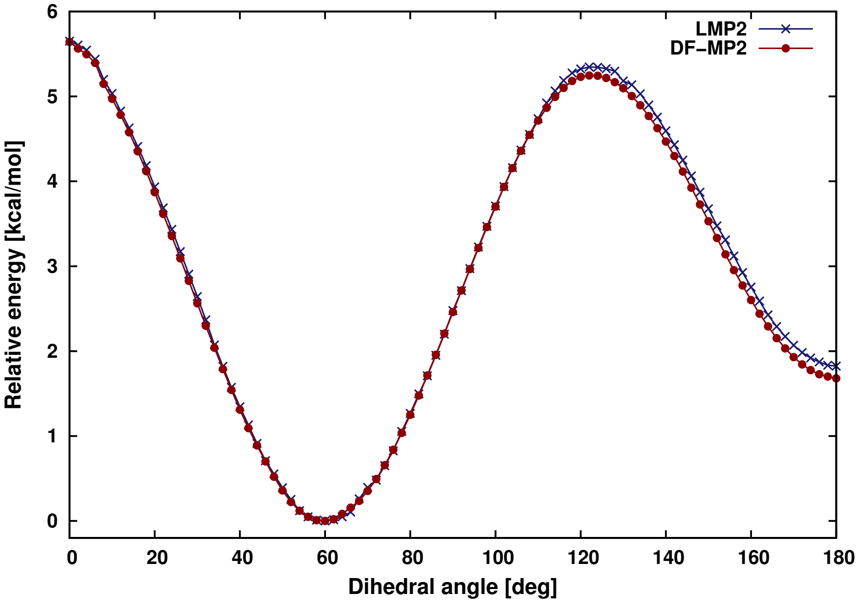

Figure S2: Rotational potential energy surface of the ethane-1,2-diphenyl radical.

References

(1) Guo, Y.; Sivalingam, K.; Valeev, E. F.; Neese, F. SparseMaps—A systematic infrastructure for reduced-scaling electronic structure methods. III. Linear-scaling multireference domain-based pair natural orbital N-electron valence perturbation theory. *J. Chem. Phys.* **2016**, *144*, 094111.

(2) Kállay, M.; Nagy, P. R.; Mester, D.; Rolik, Z.; Samu, G.; Csontos, J.; Csóka, J.; Szabó, P. B.; Gyevi-Nagy, L.; Hégely, B.; Ladjánszki, I.; Szegedy, L.; Ladóczki, B.; Petrov, K.; Farkas, M.; Mezei, P. D.; Ganyecz, Á. The MRCC program system: Accurate quantum chemistry from water to proteins. *J. Chem. Phys.* **2020**, *152*, 074107.

(3) MRCC, a quantum chemical program suite written by M. Kállay, P. R. Nagy, Z. Rolik, D. Mester, G. Samu, J. Csontos, J. Csóka, P. B. Szabó, L. Gyevi-Nagy, I. Ladjánszki, L. Szegedy, B. Ladóczki, K. Petrov, M. Farkas, P. D. Mezei, and B. Hégely. See https://www.mrcc.hu/ (Accessed Jan 1, 2021).

(4) Saitow, M.; Becker, U.; Riplinger, C.; Valeev, E. F.; Neese, F. A new near-linear scaling, efficient and accurate, open-shell domain-based local pair natural orbital coupled cluster singles and doubles theory. *J. Chem. Phys.* **2017**, *146*, 164105.

(5) Kumar, A.; Neese, F.; Valeev, E. F. Explicitly correlated coupled cluster method for accurate treatment of open-shell molecules with hundreds of atoms. *J. Chem. Phys.* **2020**, *153*, 094105.

(6) Grimme, S. Improved second-order Møller–Plesset perturbation theory by separate scaling of parallel- and antiparallel-spin pair correlation energies. *J. Chem. Phys.* **2003**, *118*, 9095.

(7) Ma, Q.; Werner, H.-J. Scalable Electron Correlation Methods. 7. Local Open-Shell Coupled-Cluster Methods Using Pair Natural Orbitals: PNO-RCCSD and PNO-UCCSD. *J. Chem. Theory Comput.* **2020**, *16*, 3135.

(8) Ghafarian Shirazi, R.; Neese, F.; Pantazis, D. A. Accurate Spin-State Energetics for Aryl Carbenes. *J. Chem. Theory Comput.* **2018**, *14*, 4733.
